# The RecBCD complex interacts directly with the DNA sliding clamp in *Escherichia coli*

**DOI:** 10.1093/nar/gkag570

**Published:** 2026-06-11

**Authors:** Ida Mathilde Marstein Riisnæs, Synnøve Brandt Ræder, Signe Simonsen, Krister Vikedal, Paul Hoff Backe, Line Johnsen, Magnar Bjørås, James Alexander Booth, Birthe B Kragelund, Kirsten Skarstad, Emily Helgesen

**Affiliations:** Department of Microbiology, Oslo University Hospital HF, Rikshospitalet, 0372 Oslo, Norway; Department of Microbiology, University of Oslo, 0316 Oslo, Norway; Department of Microbiology, Oslo University Hospital HF, Rikshospitalet, 0372 Oslo, Norway; Structural Biology and NMR Laboratory, Department of Biology, University of Copenhagen, 2200 Copenhagen, Denmark; Department of Microbiology, Oslo University Hospital HF, Rikshospitalet, 0372 Oslo, Norway; Department of Microbiology, University of Oslo, 0316 Oslo, Norway; Department of Microbiology, Oslo University Hospital HF, Rikshospitalet, 0372 Oslo, Norway; Department of Medical Biochemistry, Institute of Clinical Medicine, University of Oslo, 0372 Oslo, Norway; Department of Microbiology, Oslo University Hospital HF, Rikshospitalet, 0372 Oslo, Norway; Department of Microbiology, Oslo University Hospital HF, Rikshospitalet, 0372 Oslo, Norway; Department of Microbiology, University of Oslo, 0316 Oslo, Norway; Department of Clinical and Molecular Medicine, Norwegian University of Science and Technology, 7030 Trondheim, Norway; Department of Microbiology, Oslo University Hospital HF, Rikshospitalet, 0372 Oslo, Norway; Department of Clinical and Molecular Medicine, Norwegian University of Science and Technology, 7030 Trondheim, Norway; Structural Biology and NMR Laboratory, Department of Biology, University of Copenhagen, 2200 Copenhagen, Denmark; Department of Microbiology, Oslo University Hospital HF, Rikshospitalet, 0372 Oslo, Norway; Department of Microbiology, Oslo University Hospital HF, Rikshospitalet, 0372 Oslo, Norway; Department of Clinical and Molecular Medicine, Norwegian University of Science and Technology, 7030 Trondheim, Norway

## Abstract

DNA sliding clamps are central coordinators of genome replication and maintenance, yet the full binding network (“interactome”) of the bacterial β-clamp remains incompletely defined. Here, we report a novel interaction between *Escherichia coli* β-clamp and the helicase-nuclease RecBCD complex. Using bacterial two-hybrid assays and co-immunoprecipitation, supported by fluorescence microscopy, we show that RecB associates with β-clamp. Nuclear magnetic resonance spectroscopy maps the interaction to the canonical ligand pocket of β-clamp and identifies a clamp-binding motif in RecB (residues 1018–1023, QVEMEF), whose mutation abolishes binding. Functional assays indicate that this interaction occurs upon conformational switching of RecBCD at a Chi site, and disruption of the motif reduces survival after DNA damage. We also find indications of a second binding site in the helicase domain of RecB. These findings expand the β-clamp interactome and suggest a previously unappreciated role for β-clamp in DNA double-strand break repair, with potential implications for antibacterial strategies.

## Graphical Abstract

**Graphical Abstract:**
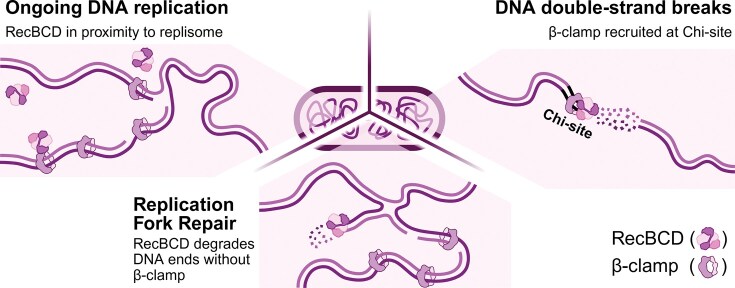

## Introduction

DNA sliding clamps are ring-shaped protein complexes fundamental to DNA replication and genome maintenance across all domains of life. In bacteria, the protein β-clamp (*dnaN*) serves as an essential interaction hub, coordinating multiple cellular processes through binding to diverse partners [1]. As a processivity factor, it ensures efficient DNA replication by tethering DNA polymerases (Pol I and the α and ε subunits of Pol III) to the DNA [2, 3]. β-clamp also engages with the replication initiation factor DnaA [4], its regulator Hda [5], as well as DNA ligase [2]. Additionally, it facilitates DNA damage tolerance and repair pathways through interactions with specialized translesion synthesis polymerases (Pol II, Pol IV, and Pol V) [6–9] and with mismatch repair proteins (MutL and MutS) [2]. Recently, β-clamp was also shown to be involved in post-replication gap repair via interaction with RecF [10].

The molecular basis for these diverse interactions lies in a conserved hydrophobic pocket present on each monomer of the homodimeric β-clamp. Partner proteins possess specific clamp-binding motifs (CBMs) that engage with these pockets [1]. Studies have demonstrated that β-clamp can simultaneously accommodate two different binding partners, with each protein occupying one pocket each [11]. The canonical CBM was initially characterized as QL[S/D]LF [12]. However, recent analyses have led to revised consensus sequences, such as Qφx[L/M][F/L] and Qφx[L/M]x[F/L], where φ represents an aliphatic residue (leucine, isoleucine, valine, or alanine) and x denotes any residue [1]. These CBMs typically span five to six residues, with distinct functional roles for specific residues. While the glutamine (Q) at the first position of the motif significantly enhances binding affinity, it is not absolutely required for interaction with β-clamp, as exemplified by the clamp loader’s δ subunit, which contains an alanine at this position [1]. Conversely, the residues at the end of the motif are critical for binding, with the leucine-phenylalanine (LF) combination providing highest affinity [1].

**Figure 1.**
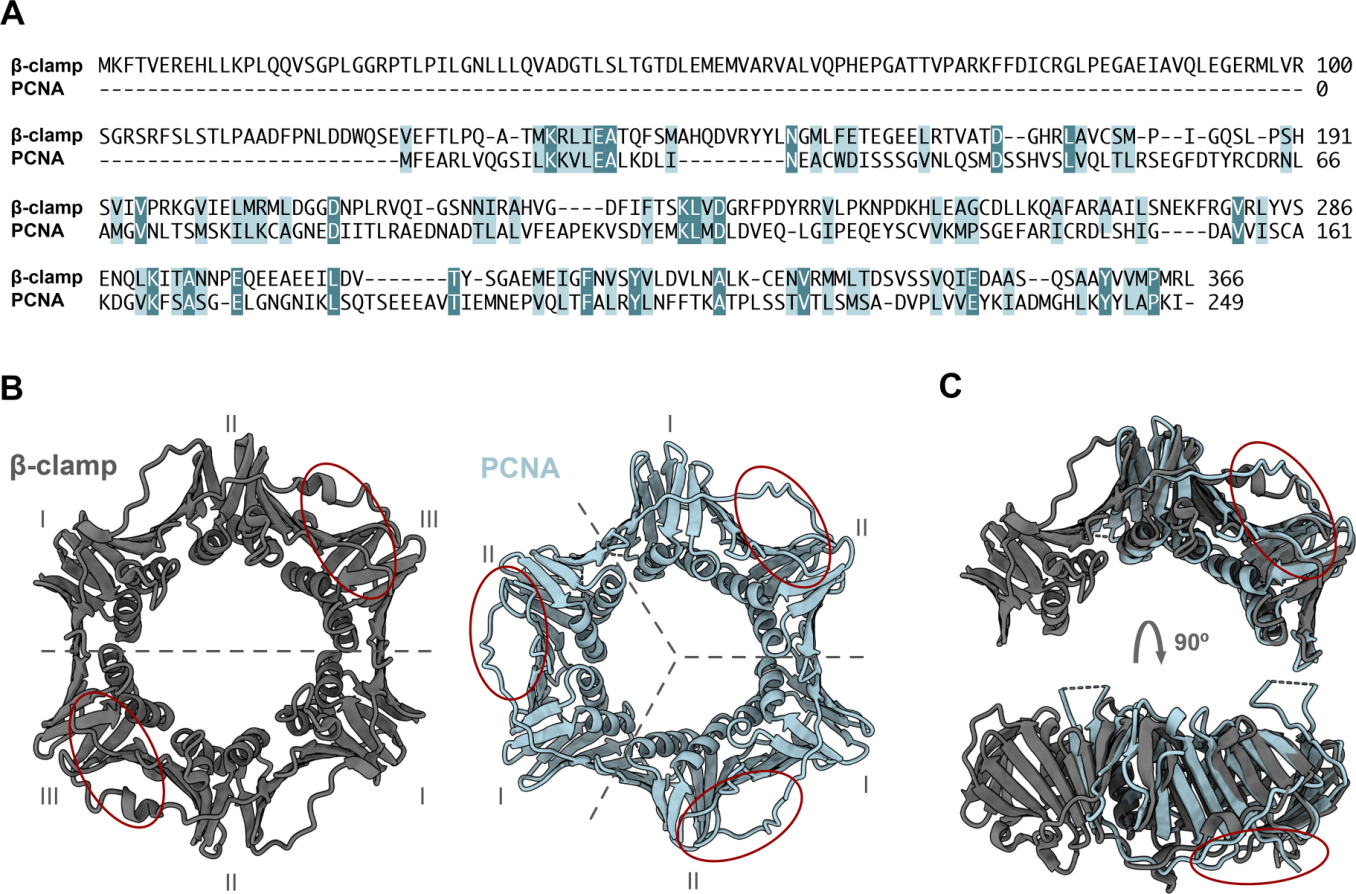
Structural conservation between β-clamp and PCNA. (**A**) Sequence alignment based on a structural alignment using the TM-align algorithm in the PDB Pairwise Structural Alignment tool. Sequences absent in the structures are thus not a part of the sequence alignment. Sequence conservation is visualized using the Color Align Conservation tool at www.bioinformatics.org. Dark green shades are identical residues, and light green shades homologous residue types. (**B**) Crystal structures of β-clamp (PDB ID 1MMI [20]; gray) and PCNA (PDB ID 1AXC [19], ligand not shown; blue). The structural domains are numbered I, II, and III (I and II for PCNA), the quaternary structure highlighted with dashed lines, and the canonical CBM binding pockets indicated with red circles. (**C**) Structural alignment of a single subunit of β-clamp (PDB ID 1MMI; gray) and PCNA (PDB ID 1AXC; blue).

The eukaryotic counterpart of β-clamp, proliferating cell nuclear antigen (PCNA), plays an analogous role in replication and repair [13]. PCNA interacts with an extensive network of >600 potential partners [14], many with direct interactions, including DNA polymerases δ/ε, FEN1 [15, 16], and numerous repair and chromatin factors [17]. This makes PCNA a prime target for developing sensitizing and antimutagenic inhibitors in cancer treatment [18]. Despite low overall sequence identity (∼11%; Fig. 1A), β-clamp and PCNA share remarkable structural conservation: both adopt closed, ring-shaped architectures encircling DNA, though β-clamp forms a homodimer of three-domain subunits, whereas PCNA forms a homotrimer of two-domain subunits (Fig. 1B) [19, 20]. Structural alignment shows that domain I–II of PCNA correspond closely to domains II–III of β-clamp (RMSD of 2.69 Å; Fig. 1C), maintaining a conserved overall topology and hydrophobic interaction pockets required for partner binding [13]. Supporting this, AlkB homologue 2 PCNA-interacting motif (APIM)-containing peptides, originally identified as PCNA-interacting motifs, also bind the hydrophobic pocket of β-clamp, providing direct evidence for the functional conservation of these interaction surfaces [21].

In eukaryotes, PCNA participates in most DNA repair pathways, including base excision repair, mismatch repair, and recombination-associated DNA synthesis through interactions with Rad51 and its mediators [22, 23]. In bacteria, by contrast, β-clamp has not been directly implicated in homologous recombination. Instead, recombination-mediated repair and double-strand break (DSB) processing are primarily executed by the RecBCD complex, composed of the RecB, RecC, and RecD subunits. RecBCD binds to double-stranded DNA (dsDNA) ends, exhibiting both helicase and nuclease activities, and facilitates RecA (the bacterial Rad51 homolog) loading onto single-stranded DNA (ssDNA) [24, 25]. Upon encountering Chi sites—8-nt DNA motifs (5′-GCTGGTGG-3′)—RecBCD transitions from indiscriminate degradation to a recombination-promoting mode, degrading the 5′ strand, while continuing to unwind and protect the 3′ strand. This transition facilitates RecA loading and promotes precise homologous recombination [26–29].

Beyond canonical DSB repair, RecBCD and RecA also play crucial roles in the restart of stalled or reversed replication forks—a frequent consequence of DNA damage or replication stress [30–32]. When replication forks collapse or regress into four-way “chicken-foot” structures, RecBCD can process the resulting DNA ends, while RecA promotes strand exchange and re-establishment of replication through homologous pairing [33–35]. Restart can also occur via RecA-independent mechanisms, involving nucleases such as RecJ or helicases like RecG and PriA [36–39]. Given that the β-clamp is already known to interact with several proteins central to replication restart, including DNA polymerases, ligase, and RecF [10], a potential link between β-clamp and the RecBCD complex could represent an additional layer of coordination between DNA replication, recombination, and repair.

Taken together, the evolutionary and functional parallels between β-clamp and PCNA, and their shared role as interaction hubs at sites of DNA synthesis and repair, raise the intriguing possibility that β-clamp might also participate in recombination-associated processes in bacteria. Here, we demonstrate that β-clamp interacts directly with RecB of the *Escherichia coli* RecBCD complex through a distinct CBM, influencing RecBCD-dependent activities.

## Materials and methods

### Strain construction and growth conditions

Strains and plasmids used in this study are listed in Tables 1 and 2, respectively. Most of the strains are derived from the adenylate cyclase-deficient (*cya*^−^) *E. coli* (reporter) strain BTH101, which is a commonly used host organism for investigation of protein–protein interactions using the bacterial two-hybrid system [40, 41]. Gene deletions or mutations were introduced into BTH101 using standard P1 transduction [42]. Single deletion mutants available in the Keio collection [43] were used to knock out *recA, recB*, or *recC*, while DPB271 was used as donor for the introduction of a *recD* mutation. Flp recombinase (pCP20) [44] was used to remove the *kan* cassette from strains transformed with plasmids carrying kanamycin resistance. Variants of pKT25 and pUT18C plasmids harboring *dnaN* and *recB* (wild type and mutants) were constructed by GenScript Inc (Piscataway, NJ, USA), except for plasmids pUT18C-recB_Δheli F1023A_ and pUT18C-*recB*_Δnuc F489A_, in which the F-to-A substitutions were generated in house using site-directed mutagenesis (see below). His-tagged *recB* was cloned into the pBAD30 vector (pBAD30-*recB*) by Dongxuan Gene Technology Co., Ltd (Guangzhou, China). Strains from frozen glycerol stock were streaked onto LB-agar plates with appropriate antibiotics and supplements for selection and incubated at 37°C, unless otherwise specified.

**Table 1.** *Escherichia coli* strains used in this study

Strain	Relevant genotype	Source
BTH101	F^−^, *cya-99, araD139, galE15, galK16, rpsL1* (*Str*^r^), *hsdR2, mcrA1, mcrB1* [Str^R^]	Euromedex BACTH system kit [45]
JW2788	BW25113 *∆recB::kan* [Kan^R^]	Keio collection [43]
IMR04	BTH101 *∆recB* [Str^R^]	This work; P1 JW2788 × BTH101 (*kan* removed with pCP20)
JW2790	BW25113 *∆recC::kan* [Kan^R^]	Keio collection [43]
IMR05	BTH101 *∆recC* [Str^R^]	This work; P1 JW2790 × BTH101 (*kan* removed with pCP20)
DPB271	λ^−^*recD1903*::mini*tet* (*recD*::mini*tet*) [Tet^R^]	Biek DP and Cohen SN [46]
IMR07	BTH101 *recD*::mini*tet* [Tet^R^]	This work; P1 DPB271 × BTH101
JW2669	BW25113 *∆recA::kan* [Kan^R^]	Keio collection [43]
IMR13	BTH101 *∆recA* [Str^R^]	This work; P1 JW2669 × BTH101 (*kan* removed with pCP20)
IMR19	BTH101 *∆recA::kan ∆recB* [Str^R^, Kan^R^]	This work; P1 JW2669 × IMR04
BHC132	MG1655 *recB::HaloTag*	Meriem El Karoui [47]
OZA001	MG1655 *gfp-dnaN frt-kan* [Kan^R^]	Tsutomu Katayama [48]
EH219	MG1655 *gfp-dnaN recB::HaloTag* [Kan^R^]	This work; P1 OZA001 × BHC132

P1 transduction is shown as: P1 Donor × Recipient. Relevant antibiotic resistances are shown in brackets: Kan^R^, Kanamycin resistance; Str^R^, Streptomycin resistance; Tet^R^, Tetracycline resistance.

**Table 2.** Main plasmids used in this study

Plasmid	Description	Mutation(s)	Source
pKT25	N-terminal T25 fusion vector [Kan^R^]		Euromedex BACTH system kit [45]
pUT18C	N-terminal T18 fusion vector [Amp^R^]		Euromedex BACTH system kit [45]
pKT25-*zip*	pKT25 containing T25::*zip* [Kan^R^]		Euromedex BACTH system kit [45]
pUT18-*zip*	pUT18C containing T18::*zip* [Amp^R^]		Euromedex BACTH system kit [45]
pKT25-*dnaN*	pKT25 containing *dnaN* [Kan^R^]		This work
pUT18C-*recB*_WT_	pUT18C containing wild type *recB* [Amp^R^]		This work
pUT18C-*recB*_Δheli_	pUT18C containing the *recB* nuclease domain [Amp^R^]	∆2–927	This work
pUT18C-*recB*_Δheli, mutQVEMEF_	pUT18C containing the *recB* nuclease domain with complete alanine substitution of ^1018^QVEMEF^1023^ [Amp^R^]	∆2–927; Q1018A / V1019A / E1020A / M1021A / E1022A / F1023A	This work
pUT18C-recB_Δheli F1023A_	pUT18C containing the *recB* nuclease domain with F-to-A substitution of residue 1023 [Amp^R^]	F1023A	This work
pUT18C-*recB*_Δnuc_	pUT18C containing the *recB* helicase domain [Amp^R^]	∆928–1180	This work
pUT18C-*recB*_Δnuc, mutQALRF_	pUT18C containing the *recB* helicase domain with complete alanine substitution of ^485^QALRF^489^ [Amp^R^]	∆928–1180; Q485A / L487A / R488A / F489A	This work
pUT18C-*recB*_Δnuc F489A_	pUT18C containing the *recB* helicase domain with F-to-A substitution of residue 489 [Amp^R^]	F489A	This work
pUT18C-*recB*_F1023A_	pUT18C containing *recB* with F1023A substitution [Amp^R^]	F1023A	This work
pUT18C-*recB*_F489A_	pUT18C containing *recB* with F489A substitution [Amp^R^]	F489A	This work
pUT18C-*recB*_F489A, F1023A_	pUT18C containing *recB* with F489A and F1023A substitutions [Amp^R^]	F489A, F1023A	This work
pKT25-*recC*_WT_	pKT25 containing wild type *recC* [Kan^R^]		This work
pKT25-*recA*_WT_	pKT25 containing wild type *recA* [Kan^R^]		This work
pBAD30	Empty plasmid [Amp^R^]		Michael T. Laub [49]
pBAD30-*recB*	pBAD30 containing his-tagged *recB* [Amp^R^]		This work
pET16b-*dnaN*	pET16b containing his-tagged *dnaN* [Cam^R^, Amp^R^]		[21, 50]

Relevant antibiotic resistances are shown in brackets: Amp^R^, Ampicillin resistance; Cam^R^, Chloramphenicol resistance; Kan^R^, Kanamycin resistance.

### Site-directed mutagenesis

Targeted mutations (F1023A and F489A) were introduced into the plasmids pUT18C-recB_Δnuc_ and pUT18C-recB_Δheli_, respectively, using the QuikChange II XL Site-Directed Mutagenesis Kit (Agilent Technologies) according to the manufacturer’s instructions. Briefly, complementary oligonucleotide primers containing the desired point mutations (Table 3) were annealed to 20 ng of template DNA. The thermal cycling program consisted of an initial denaturation at 95°C for 30 s, followed by 16 cycles of 95°C for 30 s, 60°C for 1 min, and 68°C for 8.5 min, with a final extension at 68°C for 8.5 min. Reaction mixtures were treated with *Dpn*I endonuclease for 2 h at 37°C to digest parental DNA. The products were transformed into One Shot™ TOP10 Chemically Competent *E. coli* (Invitrogen) via heat shock. The presence of the intended mutations was confirmed by DNA sequencing (Eurofins Genomics, Germany) using the primers listed in Table 3.

**Table 3.** Oligonucleotide primers for *recB* site-directed mutagenesis and sequence verification

Primer	Primer template	Sequence 5′ to 3′^a^	Length (bp)	Tm (°C)^b^
Forward mutagenesis primer 1	pUT18C-recB_Δnuc_	CAGCCGGGAAAAATCAGGCGTTACGT**GCT**GTATTTAAAGGTG	42	73.4
Reverse mutagenesis primer 1	pUT18C-recB_Δnuc_	CACCTTTAAATAC**AGC**ACGTAACGCCTGATTTTTCCCGGCTG	42	73.4
Forward mutagenesis primer 2	pUT18C-recB_Δheli_	CAGGTGGAGATGGAG**GCT**TATCTGCCGATTAGTG	34	71.9
Reverse mutagenesis primer 2	pUT18C-recB_Δheli_	CACTAATCGGCAGATA**AGC**CTCCATCTCCACCTG	34	71.9
Sequencing primer 1	pUT18C-recB_Δnuc_	GCAGGCCATATATGCATTCC	20	57.3
Sequencing primer 2	pUT18C-recB_Δheli_	CCAGCGTCGTTGAAGAAC	18	56.0

^a^Bold nucleotides indicate the introduced mutations.

^b^Tm provided by Eurofins.

### Yeast two-hybrid screening

Yeast-two hybrid screening was outsourced to Hybrigenics (Paris, France). This service employs a high-complexity *E. coli* prey library (∼3 × 10^7^ clones) and follows a robust mating-based screening protocol to identify binary protein interactions. Full-length *E. coli dnaN* (encoding β-clamp) was cloned into the Gal4 DNA-binding domain expression vector pGBKT7, which carries LEU2 and TRP1 selection markers (bait plasmid). The *dnaN* bait was screened against an *E. coli* complementary DNA/genomic prey library provided by Hybrigenics, totaling ~29 million interaction tests. Transformants were selected on medium lacking leucine, tryptophan, and histidine, supplemented with 0.5 mM of 3-amino-1,2,4-triazole (3-AT) to suppress background growth. Positive interactions were confirmed and sequenced by Hybrigenics.

### Bacterial two-hybrid (BACTH) assay

Protein–protein interactions between RecB and β-clamp, as well as between RecB and RecA, were examined using the bacterial adenylate cyclase-based two-hybrid (BACTH) system (Euromedex), as previously described [51], with optimizations noted below. This system relies on the functional reconstitution of *Bordetella pertussis* adenylate cyclase (CyaA) fragments T25 and T18, which are genetically fused to the proteins of interest. Upon interaction of the fusion partners, cAMP production is restored in the *cyaA*-deficient *E. coli* BTH101 strain, activating downstream reporters such as *lacZ* and *mal* operons. Co-transformants were tested using one or several of the following readouts: (i) qualitative X-gal spot assay, (ii) quantitative β-galactosidase assay, and (iii) semi-quantitative growth in M63 minimal medium with maltose.

#### Strain cultivation and plasmid transformation

Recipient strains (BTH101 or derivatives) were streaked from glycerol stock onto LB agar plates containing streptomycin (100 µg/ml), isopropyl β-D-1-thiogalactopyranoside (IPTG) (0.5 mM), and 5-bromo-4-chloro-3-indolyl β-D-galactopyranoside (X-gal) (40 µg/ml) and incubated at 37°C until cya⁺ (blue) revertants became visible among the cya⁻ (white) colonies. Multiple white colonies were picked and cultured overnight in LB with streptomycin (100 µg/ml) at 37°C. These cultures were diluted 1:50 and expanded before electroporation.

Plasmid constructs (typically ∼30 ng DNA) were introduced by electroporation into electrocompetent cells, which were prepared by growing to optical density at 600 nm (OD_600_) around 0.5, followed by three washes in ice-cold ultrapure water (Millipore, Milli-Q) and final resuspension in 40 µl ice-cold Milli-Q. Electroporation was carried out in 1-mm gap cuvettes (Thermo Fisher Scientific Inc., Waltham, MA, USA) at 1.35 kV, 600 Ω, and 10 µF using a Gene Pulser II (Bio-Rad). Transformed cells were immediately recovered in 1 ml SOC at 37°C for 1.5 h with shaking and plated on LB agar supplemented with appropriate antibiotics (Table 4). Plates were incubated at 30°C for up to 3 days.

**Table 4.** Antibiotics and inducers used in BACTH experiments

Additive	LB medium	M63 minimal medium
Streptomycin	100 µg/ml	50 µg/ml
Kanamycin	50 µg/ml (plasmid); 30 µg/ml (strain)	25 µg/ml (plasmid)
Ampicillin	100 µg/ml	50 µg/ml
Tetracycline	5 µg/ml*	2.5 µg/ml*
IPTG	0.5 mM	0.5 mM
X-gal	40 µg/ml	-

* Used when required for selection of specific constructs.

#### Culture conditions for assays

Following selection, 3–5 colonies from each transformation plate were pooled to inoculate 1 ml LB containing IPTG (0.5 mM) and the appropriate antibiotics. Cultures were incubated overnight at 30°C with shaking. The same overnight culture was used to seed all subsequent assays. The following day, cultures were diluted 1:100 into fresh LB (containing IPTG and antibiotics) and grown at 30°C to mid-exponential phase (OD_600_ around 0.5–0.6, measured by Nanodrop One, Thermo Fisher Scientific Inc.) before use in assays.

#### Assay I: X-gal spot assay (qualitative)

The X-gal spot assay was used to visualize protein−protein interactions via blue/white colorimetric readout. Mid-log cultures were serially diluted (10-fold series), and 5 µl of each dilution (including undiluted) was spotted onto LB agar containing IPTG (0.5 mM), X-gal (40 µg/ml), and selective antibiotics. To assess interaction stability under genotoxic stress, an identical plate was exposed to UV light (15 J/m^2^). Plates were incubated at 30°C for 2–3 days, and images were captured with a smartphone camera.

#### Assay II: β-galactosidase activity assay (quantitative)

β-galactosidase activity was quantified using a modified Miller assay adapted for 96-well format. Cultures at OD_600_ around 0.5–0.6 were measured again in a plate reader (Victor Nivo, PerkinElmer, Waltham, MA, USA) to account for instrument variability. Cells were pelleted, resuspended in PM2 buffer (70 mM Na_2_HPO_4_, 30 mM NaH_2_PO_4_, 1 mM MgSO_4_, 0.2 mM MnSO_4_, and 100 mM β-mercaptoethanol), and permeabilized with 0.01% (w/v) sodium dodecyl sulfate (SDS) and 10% (v/v) toluene for 45 min at 37°C. Supernatants were transferred in triplicate to 96-well polypropylene plates and pre-equilibrated at 30°C. The substrate o-nitrophenyl-β-D-galactoside was added to a final concentration of 0.67 mg/ml. After 30–45 min, the reaction was stopped with 200 mM Na_2_CO_3_. Absorbance at 405 nm was recorded, and β-galactosidase activity calculated in Miller Units using the following equation:


\begin{eqnarray*}{\mathrm{Miller\ Units}} &=& 1000 \times \frac{{{\mathrm{O}}{{{\mathrm{D}}}_{405}}}}{{{\mathrm{O}}{{{\mathrm{D}}}_{600}} \times {\mathrm{culture\ volume}} \times {\mathrm{reaction\ time\ [min]}}}}.\end{eqnarray*}

#### Assay III: Selective growth in M63 minimal medium with maltose (semi-quantitative)

This assay exploits the cAMP dependence of the *mal* regulon, allowing only cya^+^ cells with interacting fusion proteins to grow on maltose as sole carbon source. Cultures were first adjusted to an OD_600_ of 1 in LB and then diluted 1:100 into M63 minimal medium (2 g/l (NH_4_)_2_SO_4_, 13.6 g/l KH_2_PO_4_, 0.5 mg/l FeSO_4_·7H_2_O, 1 g/l thiamine), supplemented with 0.4% (w/v) maltose, 0.5 mM IPTG, and appropriate antibiotics. Cultures were incubated in triplicate at 30°C with shaking, and OD_600_ was measured at 24, 48, and 72 h using a plate reader. A negative control (M63-Mal without cells) was included to monitor contamination. Growth curves were visualized in GraphPad Prism (Dotmatics).

### Preparation of cell lysate for co-immunoprecipitation

His-tagged *recB* was cloned into the pBAD30 plasmid (kind gift from Prof. Michael T. Laub, MIT and Howard Hughes Medical Institute, USA) by Dongxuan Gene Technology Co. and transformed into *E. coli* MG1655. An overnight culture was diluted 1:100 into 1 l of LB broth supplemented with ampicillin (100 µg/ml). When the culture reached an OD_600_ of 0.5, expression was induced with 0.4% (w/v) L-arabinose. After 2 h of induction at 37°C, cells were harvested by centrifugation (4000 x *g*, 10 min), washed with Phosphate buffered saline (PBS), and crosslinked with 1% formaldehyde for 20 min at room temperature. Crosslinking was quenched by the addition of glycine to a final concentration of 2.5 M. The cell pellets were washed three times with PBS, flash-frozen in liquid nitrogen, and stored at −80°C.

For lysis, cell pellets were resuspended in 10 ml of lysis buffer (50 mM Tris–HCl, pH 8.0; 500 mM NaCl; 10 mM β-mercaptoethanol; 1 mg/ml lysozyme) and subjected to sonication in 3 × 15-s pulses (Vibra cell, Sonics & Materials, Inc., Newtown, CT, USA). The lysate was clarified by centrifugation at 20 000 × *g* for 20 min at 4°C, and the supernatant was collected for subsequent analysis.

### Co-immunoprecipitation

Co-immunoprecipitation was performed using Dynabeads His-Tag Isolation and Pulldown beads (Invitrogen, Thermo Fisher Scientific Inc.), following the manufacturer’s instructions. Briefly, 2 mg of total protein from the clarified lysate was diluted in 700 µl of pull-down buffer (3.25 mM sodium phosphate, pH 7.4; 70 mM NaCl; 0.01% Tween-20) and incubated with 1 mg (25 µl) of the His-tag beads. As a negative control, Dynabeads Protein A (Invitrogen) were incubated with 1 µg of mouse polyclonal IgG (Diagenode, Liège, Belgium, C15400001-15) and the same lysate.

After incubation for 30 min at 4°C with gentle rotation, beads were washed four times with wash buffer (50 mM sodium phosphate, pH 8.0; 300 mM NaCl; 0.01% (v/w) Tween-20). Proteins were eluted by incubation in 50 µl of elution buffer (wash buffer supplemented with 300 mM imidazole) for 5 min at room temperature. Beads were removed magnetically, and eluates were analyzed by SDS–polyacrylamide gel electrophoresis (PAGE).

Purified His-tagged β-clamp and RecB were expressed from *E. coli* BL21 (pET16b-dnaN) and MG1655 (pBAD30-recB), respectively. Cultures (1 l) were grown to an OD_600_ of 0.4 and induced with 0.5 mM IPTG (β-clamp) or 0.4% L-arabinose (RecB) overnight at 17°C. Cells were harvested, resuspended in lysis buffer (50 mM Tris, pH 8, 0.5 M NaCl, 10 mM β-mercaptoethanol), and lysed via sonication. Clarified lysates (40 000 × *g*, 30 min, 4°C) were incubated with Protino Ni-NTA agarose (AH Diagnostics/MACHEREY-NAGEL, Düren, Germany) for 1 h at 4°C while gently tilting. The resin was washed thrice with lysis buffer containing 20 mM imidazole, and proteins were eluted with 300 mM imidazole.

### Imaging and analysis of RecB and β-clamp colocalization

Fixed cells with endogenously tagged *recB* and *dnaN* (strain EH219) were imaged. β-clamp was N-terminally tagged with green fluorescence protein (GFP) [48] (kind gift from Tsutomu Katayama) and has been previously validated to form replication-dependent foci and to support wild-type growth [52]. RecB carried a HaloTag inserted into a surface-exposed loop after residue S47, a modification shown to retain full biological activity as assessed by growth and resistance to nalidixic acid [47] (kind gift from Meriem El Karoui). The HaloTag was labeled with Janelia Fluor 549 HaloTag ligand (JF549-HTL) (Promega Corporation, no. GA1110, Madison, WI, USA).

Strains were first grown overnight on LB-agar plates with kanamycin for selection. Then, 2 ml LB with kanamycin was inoculated with five plate colonies to create overnight cultures. The next day, overnight cultures were diluted 1:200 in fresh LB medium with kanamycin and cultured at 37°C until exponential growth (OD_600_ of 0.10). JF549-HTL (5 µM final concentration) was then added to the culture, and incubation continued for another 60 min for staining the RecB HaloTag, before washing five times with LB. After staining, samples were taken for fixation before and after 2 and 20 min of ciprofloxacin (20 ng/ml) exposure. Samples were fixed by resuspending and incubating them in PBS with 4% (v/w) formaldehyde for 60 min, followed by washing twice with PBS and resuspension in PBS for imaging.

Imaging and analysis commenced according to the procedures previously described in Vikedal *et al*. [51]. Briefly, we used a Nikon Eclipse Ti2-E inverted microscope equipped with a 60× oil objective, a CrestOptics X-Light V3 spinning disk confocal module (50:400 µm spinning disk), a Lumencor Celeste multi-line laser and two Teledyne Photometrics Kinetix sCMOS cameras. Fixed samples were transferred onto PBS-agar pads [PBS with 1% (w/v) agarose] on microscope slides within a Gene Frame (Thermo Scientific, no. AB0576), and the sample sealed with a cover slip (#1.5 thickness). Three locations were imaged for each sample to get enough cells for analysis. Three channels were used for fluorescence imaging: a transmitted light channel for cell outlines (1 ms exposure), in addition to GFP and JF549 fluorescence channels imaged with a 50:400 µm spinning disk. GFP was excited at 477 nm with emission collected between 501–521 nm (1 s exposure). JF549 was excited at 546 nm with emission collected between 580 and 610 nm (2 s exposure). Flat-field correction was applied to all images before analyses, as described previously [51]. A beta-version of MicrobeJ (beta-version 5.13p [20]) [53] was used to segment cells and detect foci of GFP–β-clamp and RecB-HaloTag/JF549 within them. The foci numbers per cell were quantified along with their colocalization. Foci were defined as colocalized if their centers (maxima) were at most one pixel apart in any direction (pixel size is 0.108 µm). The MicrobeJ template used for this analysis is available in a Zenodo repository (see “Data availability” section).

### Peptides and proteins

All peptides were purchased from TAG Copenhagen (Søborg, Denmark). Pol III C-terminal (EQVELEFD), Pol III internal (GQADMFG), RecB nuclease motif (KQVEMEFY), and RecB nuclease motif F-A (KQVEMEAY) had one additional N- and C-terminal flanking residue outside the proposed binding motif. To increase the solubility of the RecB helicase motif peptides, one C-terminal and three flanking N-terminal residues were added to this RecB motif (GKNQALRFV) and its variant motif F-A (GKNQALRAV). All peptides, except Pol III C-terminal (EQVELEFD), were N-terminally acetylated and C-terminally amidated to remove charges in the N-and C-termini, not present in the context of the full-length proteins.

Unlabeled and ^2^H, ^13^C, ^15^N-labeled *E. coli* β-clamp was expressed and purified as previously described [54]. The β-clamp concentration was determined by absorbance at 280 nm measured on a NanoDrop ND-1000 spectrophotometer (Thermo Fisher Scientific Inc.) and using an extinction coefficient calculated by the Expasy ProtParam web tool (https://web.expasy.org/protparam/) [55]. All β-clamp concentrations are listed as the protomer concentration. The peptides were readily soluble at micromolar concentration, although at high micromolar concentrations, we cannot rule out the presence of aggregates.

### Nuclear magnetic resonance spectroscopy

#### Saturation transfer difference-nuclear magnetic resonance

Saturation transfer difference (STD)-nuclear magnetic resonance (NMR) experiments were performed at 25°C on a Bruker Avance III HD 600 MHz NMR spectrometer equipped with a 5-mm QCI Cryoprobe. All samples contained 150 µM peptide with or without 1.5 µM unlabeled β-clamp in 20 mM sodium phosphate, 100 mM NaCl, 5 mM DTT, 0.3% Dimethyl sulfoxide (DMSO), 5% D_2_O, and 125 µM DSS, pH 7.4. Samples were transferred to 5-mm NMR tubes. STD experiments used a standard Bruker setup with on-resonance irradiation at 0 ppm, selectively saturating the β-clamp while avoiding direct irradiation of the peptide signals, and off-resonance irradiation at –40 ppm, where neither protein nor peptide resonances are saturated. Saturation was applied using a train of shaped pulses for 2 s at 200 Hz, and each spectrum was recorded with 1024 scans.

#### 2D TROSY-^1^H-^15^N-HSQC

2D TROSY-^1^H-^15^N-HSQC (Two-Dimensional Transverse Relaxation Optimized Spectroscopy Heteronuclear Single Quantum Coherence experiment correlating ^1^H and ^15^N nuclei) spectra were recorded of 140 µM ^2^H, ^13^C, ^15^N-labeled β-clamp with and without the addition of 700 µM RecB nuclease motif peptide (KQVEMEFY) in 20 mM sodium phosphate, 100 mM NaCl, 5 mM DTT, 5% (v/v) D_2_O, 125 µM DSS, 2% (v/v) DMSO, pH 7.4. The estimation of the RecB motif peptide stock concentration used for the NMR HSQC experiment was based on the integration of protons from the peptide compared to the integration of protons of the DSS signal, for which the concentration in the sample is known. The samples were transferred to 5-mm Shigemi tubes, and the NMR spectra were recorded at 37°C on a Bruker Avance III 750 MHz spectrometer equipped with a cryoprobe. DSS was used to reference ^1^H, and ^15^N and ^13^C were referenced indirectly using their gyromagnetic radii. The spectra were processed in Topspin (Bruker) and analyzed in CcpNmr Analysis [56]. The β-clamp assignment was acquired from BMRB under the deposition number 52 494 [54]. Chemical shift perturbations (CSPs) were calculated using the following equation [57]:


\begin{eqnarray*}
{\mathrm{CSP}} = \Delta \delta \left( {{\mathrm{ppm}}} \right) = \sqrt {{{{\left( {\Delta \delta H} \right)}}^2} + {{{\left( {0.154 \times \Delta \delta N} \right)}}^2}} .
\end{eqnarray*}

### Isothermal titration calorimetry

Unlabeled β-clamp was buffer-exchanged into 20 mM sodium phosphate, 100 mM NaCl, 1 mM tris(2-carboxyethyl)phosphine (TCEP), pH 7.4 using a 15-ml spin filter (10 000 MWCO) (Millipore, Darmstadt, Germany). DMSO was added to the β-clamp sample to a final concentration of 0.02% (v/v). RecB nuclease motif peptide (KQVEMEFY) (dissolved in 100% DMSO) was diluted into the same buffer as above, with a final DMSO concentration of 0.02% (v/v). 70.6 µM RecB nuclease motif peptide (KQVEMEFY) was added to the cell (the peptide concentration estimated by NMR as described earlier), and 278–294 µM β-clamp was added to the syringe. Peptide and protein samples were centrifuged at 20 000 × *g* at 25°C for 15 min to degas prior to loading. The isothermal titration calorimetry (ITC) experiments were repeated three times and were recorded at 25°C on a Malvern MicroCal PEAQ-ITC instrument (Malvern, Worcestershire, United Kingdom) using a stir speed of 750 rpm. The ITC data were fit to a one-site binding model using the MicroCal PEAQ-ITC Software. Reported errors are standard error of the mean (SEM) values of the three ITC replicates.

### Survival assays

The *E. coli* BTH101 *∆recB* strain (IMR04) was transformed with pUT18C plasmids encoding either wild-type RecB, the mutant RecB_F1023A_ or the mutant RecB_F489A_, as well as with the empty pUT18C vector. Non-transformed BTH101 and BTH101 *∆recB* served as positive and negative controls, respectively. All strains were cultured at 30°C to early exponential phase (OD_600_ around 0.2, measured by Nanodrop One) before treatment.

#### UV survival assay

For UV exposure, bacterial cultures were serially diluted 10-fold in LB. From each dilution, 5 µl was spotted onto four separate LB agar plates supplemented with IPTG (0.5 mM) and the appropriate antibiotics. Once droplets had dried, one plate was kept unexposed as a control, while the remaining three plates were subjected to UV irradiation at doses of 5, 15, or 30 J/m^2^ using an CL-1000 ultraviolet crosslinker (UVP). Plates were incubated overnight at 30°C, after which colony-forming units (CFUs) were counted. Survival at each UV dose was calculated as the ratio of CFUs on the UV-exposed plates to CFUs on the unexposed control plate.

#### Ciprofloxacin survival assay

To assess ciprofloxacin sensitivity, bacterial cultures at OD_600_ around 0.2 were divided into five equal subcultures and treated with ciprofloxacin at final concentrations of 0, 2, 4, 8, or 16 ng/ml. Cultures were incubated at 37°C with shaking for 2 h. Following treatment, cells were pelleted by centrifugation and resuspended in fresh LB medium to remove residual antibiotic and halt exposure. Cultures were then serially diluted 10-fold, and 5 µl of each dilution was spotted onto LB agar plates. After overnight incubation at 30°C, CFUs were counted. Survival at each ciprofloxacin concentration was calculated relative to the number of CFUs in the untreated control.

### DNA degradation assay

*Escherichia coli* cells lacking both *recA* and *recB* were transformed with pUT18C plasmids expressing either wild-type RecB, the RecB_F1023A_ mutant, the RecB_F489A_ mutant, or the empty vector. IMR13 (*ΔrecA*) served as a positive control for RecB-mediated DNA degradation, while IMR19 (*ΔrecA ΔrecB*) was used as a negative control.

Overnight cultures were diluted to an OD_600_ of 0.01 into LB medium supplemented with appropriate antibiotics and incubated with shaking at 37°C until they reached an OD_600_ of 0.2. Each culture was split into two 1 ml aliquots. One aliquot was fixed immediately in ethanol (50% final concentration) to assess DNA content prior to UV exposure. The second aliquot was pelleted, resuspended in 1× PBS, and exposed to UV light at a dose of 50 J/m^2^ (CL-1000 Ultraviolet crosslinker, UVP). Immediately after UV exposure, cells were pelleted again, resuspended in fresh LB (with antibiotics), and incubated at 37°C with shaking for 3 h to allow DNA degradation to proceed. Following the post-UV incubation, cultures were fixed in ethanol (50% final concentration) and stored at 4°C until further processing.

#### Microscopy

For imaging of DNA content, cells were stained with 5 µg/ml Hoechst 33 258 in PBS for 10 min at room temperature. Following staining, samples were washed three times with PBS to remove excess dye. To normalize for differences in cell density across samples, cell suspensions were appropriately concentrated to ensure comparable and sufficient cell numbers for imaging. For microscopy, 10 µl of each cell suspension was applied to agarose pads (1% [w/v] agarose in PBS) prepared within a Gene Frame (Thermo Fisher Scientific Inc., Cat. No. AB0576) mounted on a standard microscope slide. After drying, pads were sealed with a #1.5 thickness coverslip.

Imaging was performed on a Leica DM6000 B fluorescence microscope equipped with a 100× oil immersion objective (Leica HCX Plan Apochromat 100×, 1.40 NA, PH3 CS), a Leica EL6000 metal-halide illumination system, and a Hamamatsu C9100-14 EM-CCD camera. For each sample, six fields of view were acquired, each including both phase-contrast and fluorescence channels. Fluorescence imaging was conducted using a narrow band-pass filter set optimized for CFP (Excitation: 426–446 nm; Emission: 460–500 nm; exposure time: 30 ms). All imaging parameters—including exposure time, illumination intensity, and gain—were kept constant across all strains, treatments, and replicates to ensure quantitative comparability and minimize experimental bias.

#### Quantification of DNA degradation

Microscopy images were analyzed using the open-source software Fiji (ImageJ) [58], along with the MicrobeJ plugin (version 5.13l) [53]. Due to minor misalignment between the phase-contrast and fluorescence channels in some images, the *Channel Aligner* tool in MicrobeJ was used to adjust alignment prior to analysis. The phase-contrast channel was then used for cell detection and segmentation using the “Medial Axis” method. Detection parameters were constrained based on cell morphology, including area, length, width, circularity, curvature, sinuosity, angularity, and intensity.

To assess DNA content, the mean fluorescence intensity of each segmented cell was measured in the Hoechst (CFP) channel across six acquired images per sample. Background correction was applied by measuring the mean fluorescence intensity from cell-free regions in each image and subtracting this value from the corresponding cellular fluorescence intensities.

### Western blot

Samples, including purified RecB, β-clamp (purified as previously explained for co-immunoprecipitation), input (10 µg; 0.5%), and pull-down eluates, and samples from BTH101 cultures used in survival assays, were mixed with 1 mM dithiothreitol (DTT) and 1× NuPAGE LDS sample buffer, then heated to 75°C for 10 min. Proteins were resolved on 4%–15% Mini-PROTEAN TGX precast gels (Bio-Rad) using Tris/Glycine/SDS running buffer. SeeBlue Plus2 Pre-stained Protein Standard (Invitrogen) was used as a molecular weight marker.

Proteins were transferred onto 0.2-µm nitrocellulose membranes using the Trans-Blot Turbo Transfer System (Bio-Rad), and membranes were blocked in 5% (w/v) skim milk (Merck) in PBS-T (PBS with 0.1% Tween-20) for 45–60 min at room temperature. Membranes showing RecB levels in BTH101 cultures were additionally blocked with cell extracts from *ΔrecB* cells. These cell extracts were made as previously explained [51] with some alterations: an anti-RecB polyclonal antibody used (rabbit, MyBiosource, cat. no. MBS7162389), and no ciprofloxacin added. Following protein transfer, these membranes were also incubated in 0.1% (w/v) Ponceau S solution for 5 min to verify transfer efficiency and account for total protein loading. The stain was visualized, imaged for densitometric normalization, and subsequently removed by washing with deionized water prior to blocking.

Membranes were incubated overnight at 4°C with primary antibodies diluted in blocking buffer: anti-RecB at 1:200, and anti-β-clamp antibody (produced to order; rabbit, Eurogentec, Seraing, Liège, Belgium) at 1:20 000. After washing, membranes were incubated for 45 min with goat anti-rabbit IgG (H + L), HRP-conjugated secondary antibody (MyBiosource, cat. no. MBS705310), followed by detection using SuperSignal West Femto Maximum Sensitivity Substrate (Thermo Fisher Scientific, cat. no. 34577). Chemiluminescent signals were visualized using the ChemiDoc MP Imaging System and Image Lab software (Bio-Rad).

Relative RecB levels were quantified from the western blot images using Fiji (ImageJ) [58]. The gel lane selection tool was used to select the regions with RecB protein (134 kDa) for each sample lane. The lane profiles were plotted, and the measured signal from each lane was first normalized to the relevant Ponceau stain signal from the same lane and then to the wild-type strain.

### Statistical analysis

Quantitative data were analyzed and plotted using GraphPad Prism (version 10.4.1). For statistical comparisons of β-galactosidase activities, ordinary one-way analysis of variance (ANOVA) with Dunnett’s correction was applied to adjust for multiple comparisons to the wild-type control. Ordinary one-way ANOVA with Šidák’s correction was used for DNA degradation results to adjust for multiple comparisons between a predefined subset of strain pairs. Comparisons of RecB–β-clamp colocalization before and after ciprofloxacin exposure were analyzed using repeated measures one-way ANOVA with Tukey’s correction to adjust for multiple comparisons between all pairs of strains. Asterisks denote the level of statistical significance. Two-tailed *P*-values below .05 were considered significant and indicated with asterisks as follows: *P ≤ .05; **P ≤ .01; ***P ≤ .001; ****P ≤ .0001.

## Results

### RecBCD interacts with β-clamp

Given the high structural conservation between bacterial and eukaryotic sliding clamps [12] and the large number of interaction partners of the eukaryotic sliding clamps [14], it is plausible that many partners of β-clamp (DnaN) remain to be discovered. The sliding clamp has proven essential for multiple repair mechanisms in eukaryotes, including repair by homologous recombination. An initial yeast two-hybrid screening with β-clamp as bait indicated a possible interaction between β-clamp and the RecB subunit of the RecBCD repair complex (Supplementary Table S1). We therefore further explored this possible interaction to decipher whether β-clamp is also involved in homologous recombination in bacteria.

We employed a bacterial two-hybrid (BACTH) system to verify the RecB–β-clamp interaction from the yeast two-hybrid screening [40, 41]. The *recB* gene was cloned into a pUT18C vector, while *dnaN* was cloned into a pKT25 vector. These constructs were co-transformed into the *E. coli* reporter strain BTH101, which lacks endogenous adenylate cyclase (*cyaA*). In addition, the constructs were co-transformed into BTH101 variant strains with single-gene deletions of *recA, recB, recC*, or *recD* to assess the interaction’s reliance on RecA or RecBCD complex formation.

**Figure 2.**
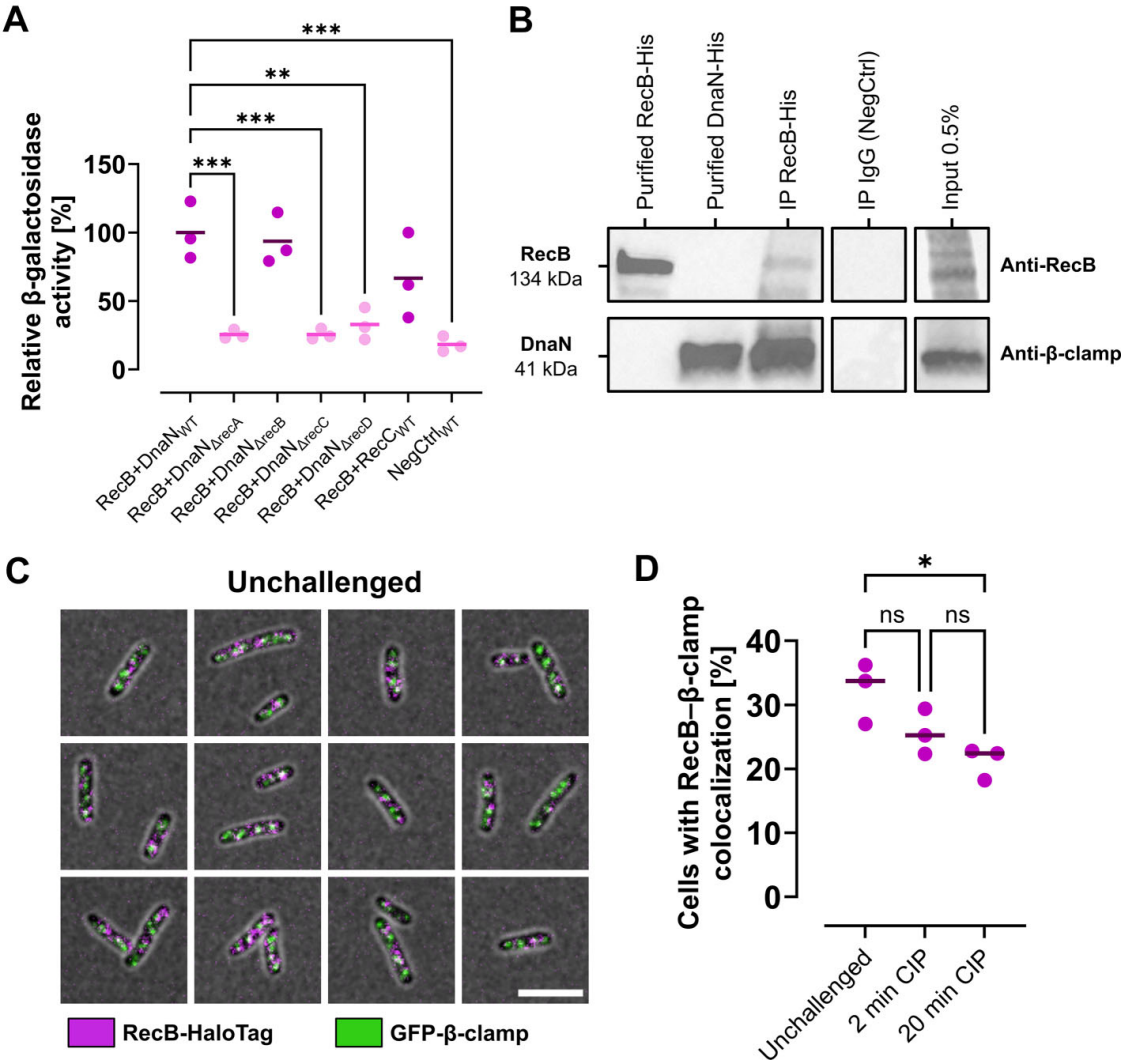
Experimental evidence for interaction between RecB and β-clamp. (**A**) Relative β-galactosidase activity from bacterial two-hybrid assay with co-expressed RecB–T18 and β-clamp (DnaN)–T25 fusion proteins in BTH101 wild type compared to *ΔrecA, ΔrecB, ΔrecC*, and *ΔrecD* deletion strains. Activities were normalized to the wild-type mean. Wild-type cells co-expressing RecB and RecC served as positive controls; negative controls used empty vectors. Strains with significantly different activity from RecB–β-clamp-expressing wild type appear in light magenta; others in dark magenta. Only significant differences are annotated. (**B**) Western blots from a co-immunoprecipitation experiment where β-clamp (DnaN) was pulled down (lower panel, anti-β-clamp) together with his-tagged RecB expressed from a pBAD30 plasmid (upper panel, anti-RecB) in an MG1655 background. (**C**) Representative images of fixed, unchallenged EH219 cells displaying fluorescence from JF549-labeled RecB-HaloTag (magenta) and endogenously expressed GFP-β-clamp (green); overlapping signal appears white. (**D**) Percentage of EH219 cells showing RecB–β-clamp colocalization when unchallenged and after 2 and 20 min of exposure to 20 ng/ml ciprofloxacin (CIP). Foci were considered colocalizing if they were at most one pixel apart in any direction (pixel size is 0.108 µm). Comparisons were made using (A) ordinary one-way ANOVA with Dunnett correction and (D) repeated measures one-way ANOVA with Tukey correction. Lines represent means from three biological replicates; dots indicate individual replicates. ns, non-significant; *P ≤ .05; **P ≤ .01; ***P ≤ .001.

We observed a distinct interaction between RecB and β-clamp using the BACTH assay, as indicated by elevated β-galactosidase activity after 24 h in both wild-type and *ΔrecB* BTH101 backgrounds, relative to the empty vector negative control strain (Fig. 2A). The strength of the interaction was comparable to the RecB–RecC positive control interaction (Fig. 2A). Notably, the interaction between RecB and β-clamp was abolished in BTH101 strains lacking either RecC or RecD, suggesting that the RecBCD complex interacts with β-clamp as a functional unit. The interaction initially appeared absent in the *ΔrecA* strain (Fig. 2A). However, when using an M63/maltose-based variant of the assay—which is less sensitive to differences in bacterial growth rates and allows detection of weaker or delayed interactions—the interaction was apparent also in the *ΔrecA* strain (Supplementary Fig. S1). This indicates that the RecB–β-clamp interaction likely persists in the absence of RecA, but that the slower growth rate of the *ΔrecA* strain necessitates longer incubation to achieve a detectable interaction (Supplementary Fig. S1).

To further validate the RecB–β-clamp interaction, we performed co-immunoprecipitation experiments using a N-terminally his-tagged (6× histidine residues) version of RecB as bait, expressed from a pBAD30 vector. Indeed, β-clamp was pulled down and exhibited a strong band on the membrane (Fig. 2B).

Fluorescence microscopy was used to examine RecB localization relative to β-clamp in a strain expressing endogenous GFP-tagged β-clamp (*gfp-dnaN)* [48] and RecB with a HaloTag [47]. After growth to exponential phase, RecB-HaloTag was labeled with JF549-HTL dye. One unchallenged sample was fixed, while the remaining culture was treated with ciprofloxacin at the minimum inhibitory concentration (20 ng/ml) to induce DNA DSBs. Samples were fixed after 2 and 20 min of exposure to assess RecB–β-clamp proximity during the DNA damage response. RecB exhibited weaker foci compared to GFP-β-clamp, along with a diffuse cytoplasmic signal. A recent publication suggests that persistent RecB foci likely represent DNA-bound RecB complexes [59]. Accordingly, images were processed to reduce the diffuse background while preserving the clear RecB foci (representative cells shown in Fig. 2C).

Quantitative colocalization analysis showed that ~30% of cells exhibited RecB and β-clamp foci within a 1-pixel distance (0.108 µm) of each other under unchallenged conditions (Fig. 2D). Given the diffraction-limited resolution of fluorescence microscopy, this distance reflects only coarse spatial proximity; it cannot by itself demonstrate a physical interaction, but it can support the possibility of a direct or closely associated interaction when interpreted together with other biochemical evidence. Under unchallenged conditions, β-clamp is known to primarily localize to active replication forks and also lags behind the replication forks on newly synthesized sister chromatids due to delayed unloading [60]. RecBCD plays a well-documented role in maintaining faithful DNA replication in situations where the replication forks stall or collapse at endogenous obstacles, such as secondary DNA structures or tightly bound proteins [61, 62]. In *E. coli*, fork stalling is estimated to occur up to five times per replication cycle (reviewed by Michel & Sandler [63]) and has been reported to result in spontaneous DSBs in up to 18% of cells at each generation [64]. Under normal growth conditions, RecBCD may therefore localize near replication forks either in a standby state or actively engaged in resolving impediments to fork progression.

After 20 min of ciprofloxacin treatment, the fraction of cells showing RecB–β-clamp colocalization decreased (Fig. 2D and Supplementary Fig. S2A). This reduction coincided with a marked decrease in the average number of β-clamp foci per cell (Supplementary Fig. S2B), likely reflecting replisome disassembly caused by the roadblock effect of ciprofloxacin-induced DNA lesions [65]. The remaining β-clamp foci after ciprofloxacin treatment may reflect clamps retained on the DNA after replisome disassembly. The number of RecB foci remained largely unchanged, in accordance with previous reports [66, 67] (Supplementary Fig. S2C). The observed reduction in colocalization can therefore not be used to determine the context in which interaction is most functionally relevant—whether during normal replication or in response to DNA DSBs, or both. Interestingly, UV damage (15 J/m^2^) appeared to enhance the RecB–β-clamp interaction, as assessed by BACTH analysis (Supplementary Fig. S3), suggesting that DNA damage context and type may differentially influence the interaction dynamics. Taken together, our results from the BACTH assay, co-immunoprecipitation, and fluorescence microscopy provide compelling indications of a previously uncharacterized interaction between the RecBCD complex and β-clamp.

### Identification of the QVEMEF motif in RecB as a functional clamp-binding motif

**Figure 3.**
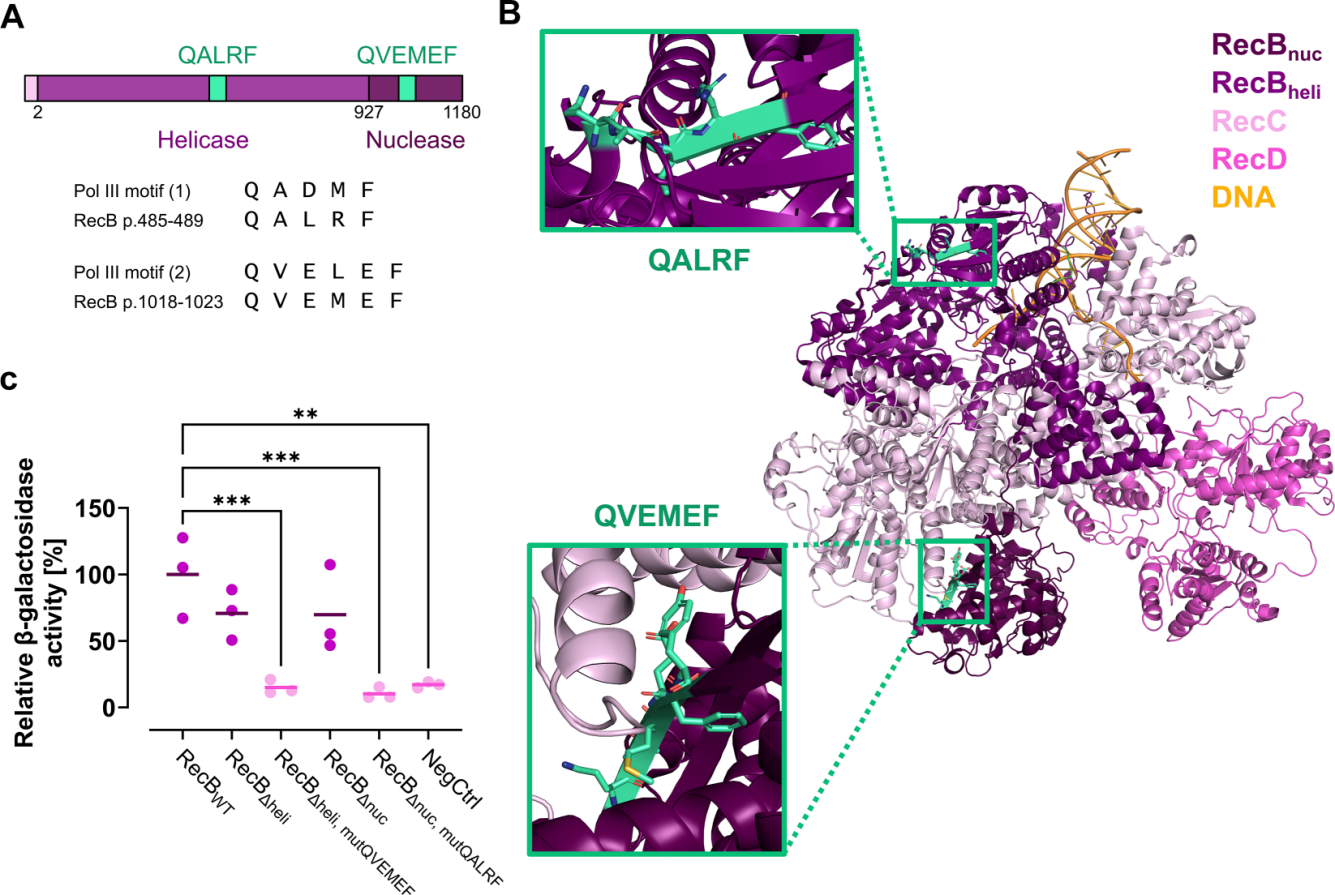
RecB may possess one CBM in each domain. (**A**) Sequence alignment showing two candidate CBMs (green) in RecB: QALRF (residues 485–489) in the helicase domain and QVEMEF (residues 1018–1023) in the nuclease domain. Both motifs exhibit sequence similarity to known CBMs from DNA polymerase III. (**B**) Structural mapping of the candidate motifs (green) within the RecBCD complex. QALRF is surface-exposed; QVEMEF is positioned near the RecC interface within a tunnel structure. Structure rendered from PDB ID 1W36 [69] using PyMOL (by Schrödinger). (**C**) Relative β-galactosidase activity from bacterial two-hybrid assay of BTH101 wild type co-expressing β-clamp–T25 and various RecB–T18 variants—full-length, truncated, and mutated. RecB_Δnuc_ includes residues 1–927; RecB_Δheli_ includes residues 928–1180. mutQALRF and mutQVEMEF indicate complete alanine substitutions of candidate motifs. Negative controls used empty vectors. Strains with significantly different activity from full-length RecB–β-clamp-expressing wild type appear in light magenta; others in dark magenta. Only significant differences are annotated. Comparisons were made using ordinary one-way ANOVA with Dunnett correction. Lines represent means from three biological replicates; dots indicate individual replicates. **P ≤ .01; ***P ≤ .001.

To further characterize the RecB–β-clamp interaction, we searched RecB’s amino acid sequence for potential CBMs. We uncovered two candidate motifs: ^485^QALRF^489^ in the helicase domain, and ^1018^QVEMEF^1023^ in the nuclease domain. These motifs share significant homology with known DNA polymerase III (Pol III) motifs [68], differing by only two or one amino acid from the Pol III CBMs QADMF and QVELEF, respectively (Fig. 3A). Investigation into the localization of the two motifs in the protein complex [69] revealed that while ^485^QALRF^489^ is surface-exposed and readily accessible, ^1018^QVEMEF^1023^ is positioned internally near the RecC protein interface in a tunnel through which the DNA protrudes, potentially affecting its availability for β-clamp binding (Fig. 3B).

To evaluate whether our predicted CBMs in the RecB helicase and nuclease domains are functional, we constructed two truncated variants of *recB* in the pUT18C vector. The first encodes RecB residues 928–1180, thereby lacking the helicase domain of RecB, and is referred to as RecB_Δheli_. The second encodes residues 1–927, excluding the nuclease domain, and is designated RecB_Δnuc_. Using the BACTH assay, we found that both truncated variants—RecB_Δheli_ and RecB_Δnuc_—retained interaction with β-clamp (Fig. 3C). These results suggest that at least one functional CBM may be present in each domain.

To assess the contribution of specific candidate motifs, we substituted each motif entirely with alanines. In RecB_Δnuc_, the candidate QALRF motif was replaced with alanines (mutQALRF), while in RecB_Δheli_, the QVEMEF motif was similarly mutated (mutQVEMEF). These domain-specific mutations were introduced into truncated RecB variants to test each motif independently, based on the rationale that potential effects might be masked in the full-length protein due to functional compensation by the other motif.

Strikingly, BACTH analysis showed that the interaction with β-clamp was abolished in both RecB_Δnuc mutQALRF_ and RecB_Δheli mutQVEMEF_ constructs, indicating that β-clamp can interact with each domain of RecB through the identified CBMs (Fig. 3C). However, the effect could also be caused by large perturbations of the protein due to full-motif substitutions. We therefore tested single amino acid substitutions of the terminal phenylalanines in these motifs, F489A and F1023A, respectively. Blue/white BACTH screening indicated that single mutations were sufficient to perturb the interaction (Supplementary Fig. S4)

To further characterize the putative CBMs in RecB, we performed NMR experiments using recombinantly expressed and purified β-clamp and synthetic peptides representing the candidate CBMs. Each peptide included the core motif flanked by one or several native residues to mimic its native sequence context. Two peptides derived from the established CBMs of *E. coli* Pol III, QVELEF and QADMF, were included as positive controls. Samples containing peptides, but no β-clamp, were used as negative controls (Supplementary Fig. S5).

**Figure 4.**
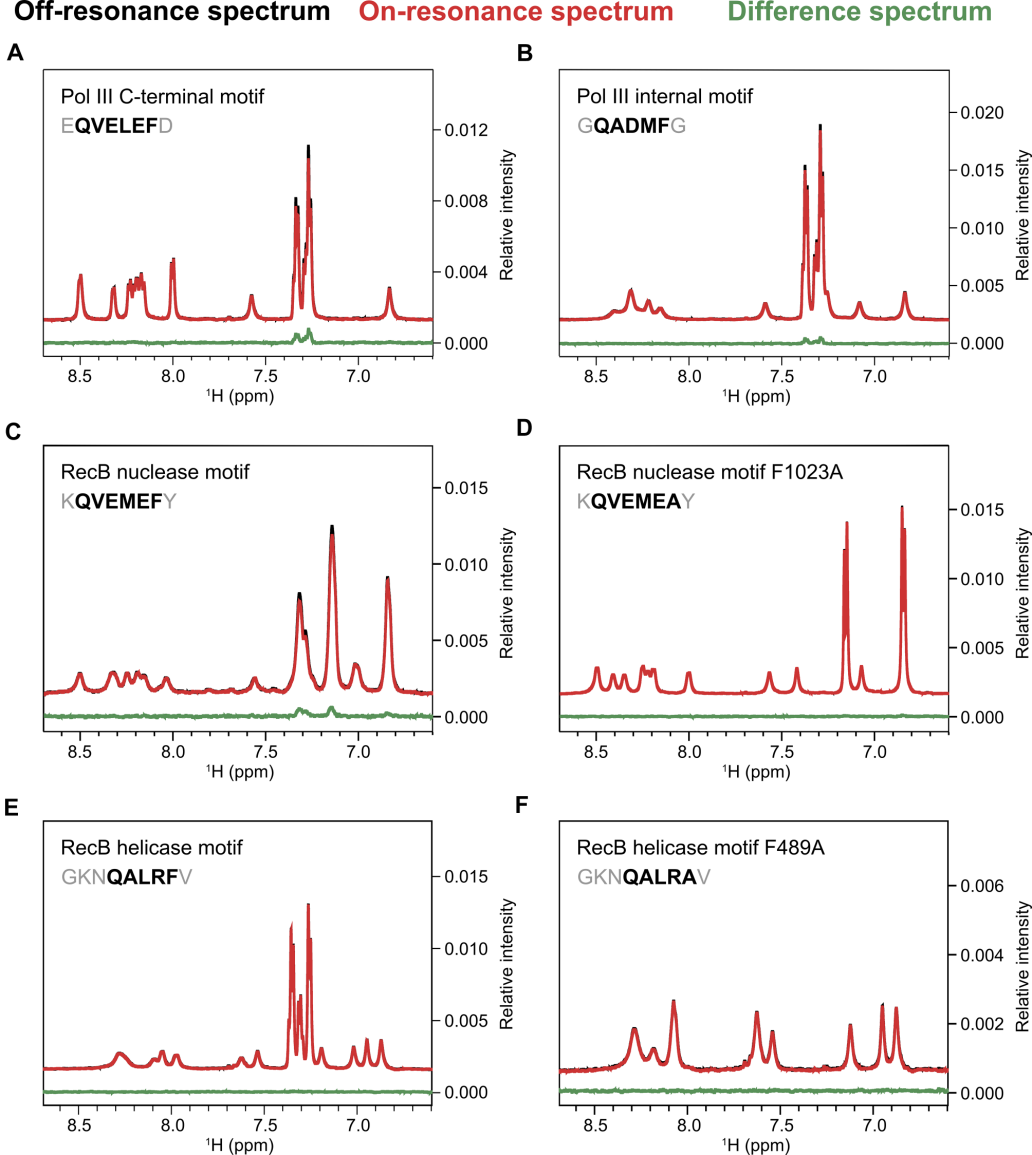
NMR analysis of β-clamp interactions with candidate CBMs from RecB. (A–F) STD-NMR spectra of synthetic peptides incubated with 1% (molar ratio) purified β-clamp protein. For each sample, the off-resonance (reference) spectrum is shown in black, the on-resonance (saturated) spectrum in red, and the STD spectrum in green. Peaks in the difference spectrum indicate transfer of saturation from β-clamp to the peptide, consistent with binding. (**A**) QVELEF and (**B**) QADMF peptides from DNA polymerase III show clear binding and serve as positive controls. (**C**) QVEMEF peptide derived from RecB shows a distinct STD signal, indicating specific interaction. (**D**) A QVEMEF variant in which the terminal phenylalanine was replaced with alanine (QVEMEA) showed no detectable binding. (**E**) QALRF peptide from RecB and (**F**) its phenylalanine-to-alanine variant (QALRA) did not exhibit detectable STD signals under the conditions tested. Peptide-only controls (no β-clamp) are shown in Supplementary Fig. S5. NMR, nuclear magnetic resonance; STD, saturation transfer difference.

STD NMR was used to assess binding interactions, where a difference in peak intensities between on-resonance and off-resonance spectra indicates ligand binding. As expected, both Pol III-derived peptides showed clear binding to β-clamp (Fig. 4A and B). Notably, the RecB-derived QVEMEF peptide also showed binding (Fig. 4C), supporting its role as a functional CBM. In contrast, a mutated QVEMEF variant in which the terminal phenylalanine was substituted with alanine (QVEMEA) showed no detectable binding (Fig. 4D), confirming our previous BACTH indication (Supplementary Fig. S4) and underscoring the critical role of this conserved aromatic residue in β-clamp interaction.

We also tested the second RecB motif, QALRF, and a corresponding F-to-A variant. Neither peptide showed detectable binding to β-clamp under the conditions tested (Fig. 4E and F, respectively). Although the QALRF motif is more surface-exposed in the RecBCD complex than the QVEMEF motif (Fig. 3B), its sequence deviates from the canonical CBM consensus and may therefore lack features necessary for interaction with β-clamp [1].

**Figure 5.**
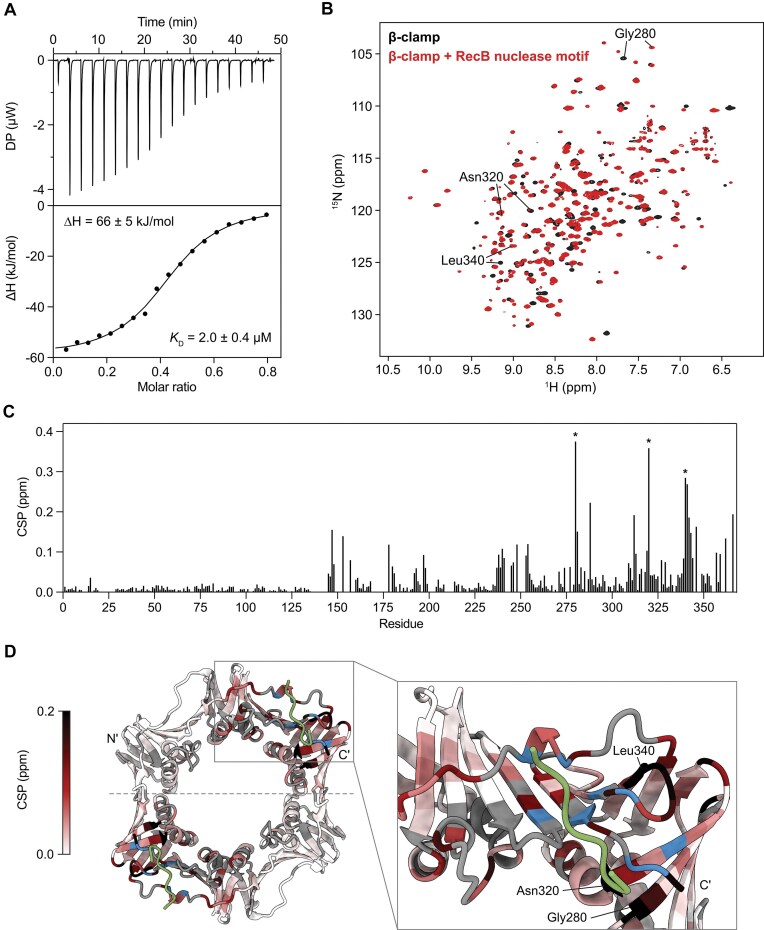
QVEMEF binds β-clamp with micromolar affinity at the canonical binding site. (**A**) ITC analysis of the interaction between the RecB-derived QVEMEF peptide and β-clamp. The upper panel shows baseline-corrected raw heat changes upon titration of β-clamp into the peptide solution; the lower panel displays the integrated binding isotherm fitted using a one-site binding model. The dissociation constant (*K*_D_) and enthalpy change (Δ*H*) represent the mean of three replicates; error bars indicate the SEM. (**B**) Overlaid ^1^H,^15^N TROSY-HSQC spectra of uniformly labeled ^2^H,^13^C,^15^N-labeled β-clamp in the absence (black) and presence (red) of a five-fold molar excess of the QVEMEF peptide. (**C**) CSPs observed upon peptide titration. Asterisks indicate residues with notable CSPs, corresponding to labeled peaks in panel (B) and mapped structurally in panel (D). (**D**) Mapping of affected residues onto the β-clamp structure, bound to the C-terminal Pol III peptide (green) (PDB ID: 3D1F). Residues showing the largest CSPs are labeled, and those that disappear during titration—indicative of intermediate exchange—are shown in blue (Leu177, Tyr244, Val247, Ile317, Ser345, Gln348, Val361, Met364, and Arg365). Unassigned residues (including prolines) are labeled gray. DP, differential power; ITC, isothermal titration calorimetry; ^1^H,^15^N TROSY-HSQC, two-dimensional transverse relaxation optimized spectroscopy heteronuclear single quantum coherence experiment correlating ^1^H and ^15^N nuclei.

To quantify the binding affinity of the QVEMEF motif, we performed ITC. The interaction yielded a dissociation constant (*K*_D_) of 2.0 ± 0.4 µM (Fig. 5A), comparable to affinities reported for other canonical CBMs [1]. Accurate determination of peptide concentration was complicated by the absence of aromatic that, combined with oxidation of methionines in the binding pocked as previously reported [54], lead to an *N*-value of 0.39 ± 0.02. However, since β-clamp was titrated from the syringe into the peptide solution in the sample cell, inaccuracies in peptide concentration affect only the *N*-value and not the calculated *K*_D_.

To confirm whether QVEMEF engages the canonical binding site pocket on β-clamp, we performed two-dimensional NMR studies. We recorded ^1^H,^15^N TROSY-HSQC spectra of ^2^H,^13^C, ^15^N-triple-labeled β-clamp in the presence and absence of the QVEMEF peptide (Fig. 5B) (See “Materials and methods” section for details). A titration series was conducted to monitor CSPs (Supplementary Fig. S6), which revealed widespread peak movements across the β-clamp sequence (Fig. 5C). This pattern, consistent with that observed for other CBMs, suggests conformational changes and altered dynamics upon ligand binding [54]. The largest CSPs localized to residues surrounding the canonical CBM binding pocket, including L177, Y244, V247, I317, S345, Q348, V361, M364, and R365. These residues became untraceable during titration due to intermediate exchange on the NMR timescale, indicating their proximity to the binding interface (Fig. 5D).

Together, these data demonstrate that the QVEMEF motif in RecB binds specifically to β-clamp at the canonical CBM binding site with an affinity typical of other known CBMs. However, contribution of QALRF cannot be excluded, despite being undetectable by NMR.

### Mutating the QVEMEF CBM (F1023A) reduces survival after DNA damage

**Figure 6.**
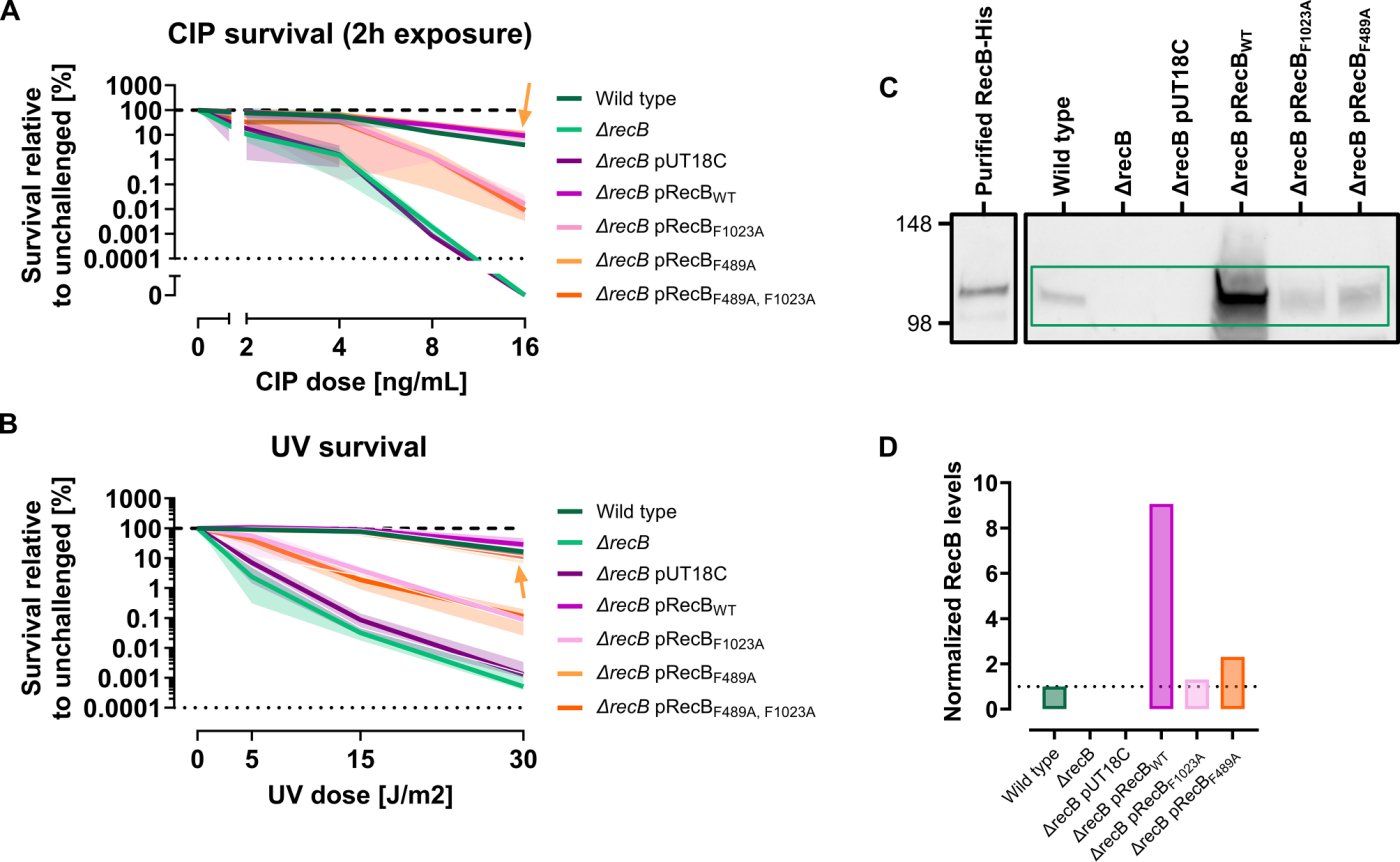
Mutation of the RecB QVEMEF CBM impairs survival following DNA damage. Survival of BTH101 *ΔrecB* cells expressing wild-type RecB (RecB_WT_) or F1023A-mutated RecB (RecB_F1023A_) from pUT18C plasmids after varying doses of (**A**) ciprofloxacin (CIP) exposure (0, 2, 4, 8, or 16 ng/ml) for 2 h or (**B**) UV irradiation (0, 5, 15, or 30 J/m^2^). Wild-type BTH101, as well as *ΔrecB* strains with or without empty pUT18C vectors, served as controls. Relative survival was calculated through comparison with unchallenged parallels. Solid lines represent means from 2 to 3 biological replicates; shaded regions indicate standard deviation. Thick dashed lines show survival of unchallenged parallels; thin dotted lines mark the detection limit. (**C**) Western blot analysis of RecB expression from pUT18C constructs in the BTH101 *ΔrecB* background compared to in wild-type background. Each well was loaded with 80 µg protein extract. The two panels shown were cropped from the same gel and processed identically. (**D**) Quantification of RecB expression from the western blot in (**C**), normalized to the loading control (Supplementary Fig. S7) and relative to RecB expression in BTH101 wild-type cells.

Having established that the QVEMEF CBM of RecB can bind to β-clamp, we next assessed the functional significance of this interaction for *E. coli* survival after DNA damage. Plasmid complementation was performed in a *ΔrecB* background using pUT18C constructs expressing either wild-type RecB (RecB_WT_) or RecB carrying the F1023A substitution in QVEMEF (RecB_F1023A_), a mutation that abolishes binding in BACTH and NMR studies (Supplementary Fig. S4 and Fig. 4D). We also included RecB with an F489A substitution in the QALRF motif, despite its lack of detectable binding in NMR studies. Expression was induced using IPTG (0.5 mM). The cells were challenged with increasing doses of ciprofloxacin or UV. We found that expressing RecB_WT_ fully restores *ΔrecB* strain survival to the wild-type strain (BTH101) level at all doses of ciprofloxacin (Fig. 6A) or UV (Fig. 6B) tested. In contrast, cells expressing RecB_F1023A_ exhibited a 100-fold reduction in survival following treatment with either 16 ng/ml ciprofloxacin or 30 J/m^2^ UV irradiation (Fig. 6A and B, respectively). The F489A mutation, on the other hand, had no effect on survival in this experimental setup and complemented survival as wild type RecB (Fig. 6A and B).

To ensure that the decreased survival was not caused by dramatic changes in the protein level of RecB_F1023A_ compared to endogenous RecB, we performed western blotting to quantify the levels of RecB expressed from the different plasmid derivatives compared to the wild-type background. Ponceau S staining of total protein was used for normalization of the data (Supplementary Fig. S7). We found that the level of mutated variants of RecB (RecB_F1023A_ and RecB_F489A_) was ∼1 and 2 times that of endogenous RecB, respectively, whereas the level expressed from pRecB_WT_ was more than eight times higher than in the plasmid-free wild-type strain (Fig. 6C and D). Since both the plasmid-free wild-type strain and the strain expressing RecB_WT_ or RecB_F489A_ exhibited comparable survival (Fig. 6A and B), the reduced survival of the RecB_F1023A_ strain is likely not caused by too low levels of RecB.

**Figure 7.**
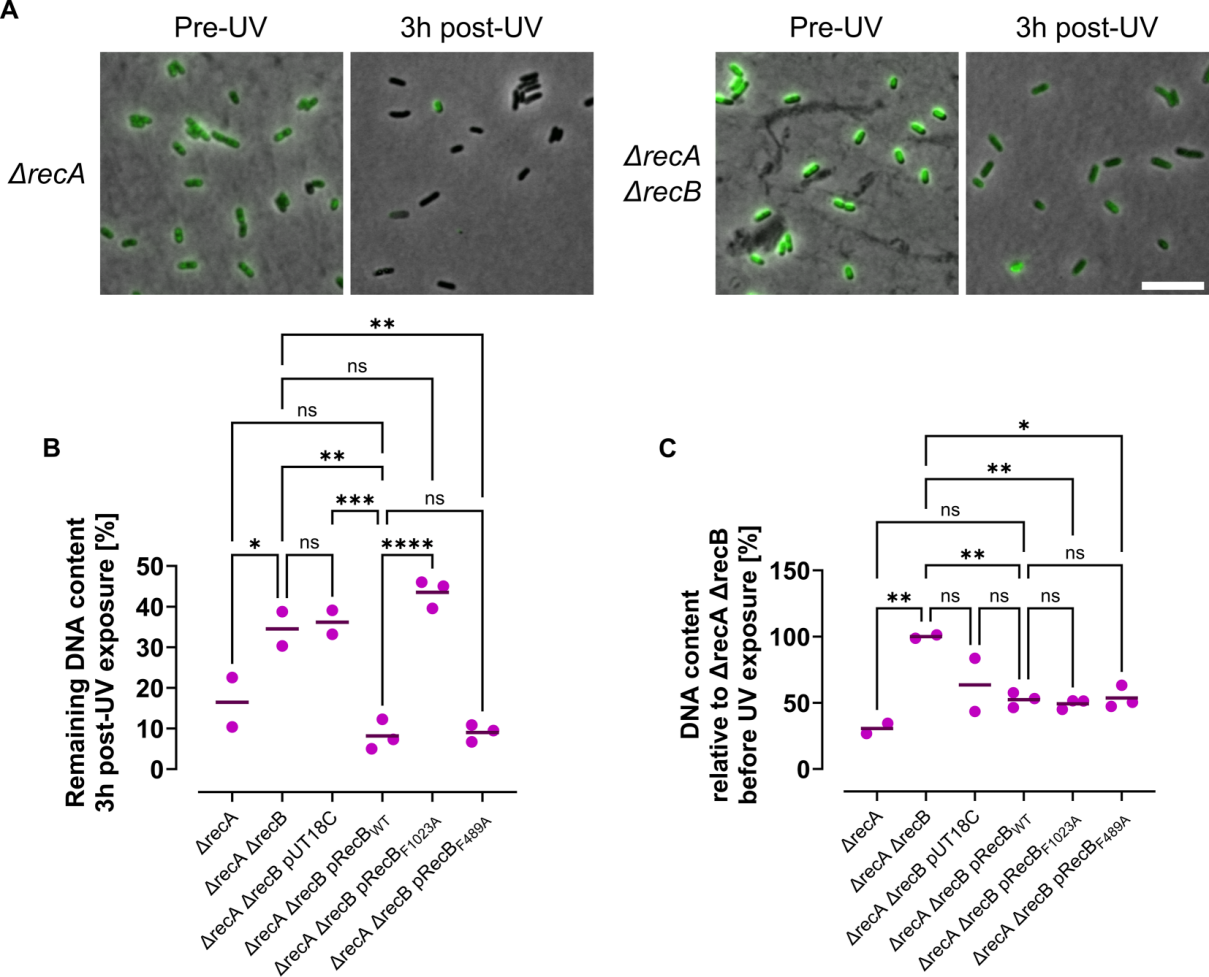
The RecB F1023A CBM mutation impairs UV-induced “reckless” DNA degradation but does not alter basal DNA content. (**A**) Representative images of Hoechst 33258-stained (green) *ΔrecA* and *ΔrecA ΔrecB* cells, fixed either before or 3 h after UV irradiation (50 J/m^2^). (**B**) Quantification of remaining DNA content following UV exposure, normalized to unchallenged controls. *ΔrecA ΔrecB* cells were complemented with either wild-type RecB (pRecB_WT_​) or with the F1023A mutant (pRecB_F1023A_) or F489A mutant (pRecB_F489A_) expressed from pUT18C plasmids; empty pUT18C vectors served as controls. (**C**) Basal DNA content under non-damaging conditions, shown relative to *ΔrecA ΔrecB* cells prior to UV exposure. Complementation was performed as in panel (B). No significant difference in DNA content was observed between RecB_WT_​ and RecB_F1023A_ or RecB_F489A_ in the absence of UV-induced damage. For all quantifications, comparisons were made using ordinary one-way ANOVA with Šidák correction. Lines represent normalized medians from 2 to 3 biological replicates; dots indicate individual replicates. ns, non-significant; *P ≤ .05; **P ≤ .01; ***P ≤ .001.

We find it unlikely that the effect of the F1023A mutation arises from abolished complex formation caused by the mutation, as this would be expected to yield a survival profile comparable to that of the *ΔrecB* strain. Importantly, BACTH analysis revealed that the interaction between RecB_F1023A_ and RecA is fully intact compared to RecB_WT_ (Supplementary Fig. S8), arguing against impaired RecA recruitment as an explanation for the survival defect. Whether the mutation could subtly affect RecB–RecC interaction is addressed below (Fig. 7C).

### The F1023A CBM mutation compromises RecBCD function in “reckless” DNA degradation after UV-induced damage

RecBCD plays a dual role in bacterial DNA metabolism (reviewed in [28, 70, 71]). Upon binding to a dsDNA end, RecBCD initiates processive DNA unwinding and degradation, acting as a potent helicase-nuclease complex. When encountering a Chi site (5′-GCTGGTGG-3′), its activity shifts: degradation is halted, a 3′ overhang is generated, and RecBCD promotes homologous recombination by facilitating RecA loading onto the ssDNA [26–28]. This molecular switch—from degradation to recombination—is central to DNA DSB repair in *E. coli*. However, in the absence of RecA, this switch cannot occur. As a result, RecBCD continues to degrade DNA unchecked, leading to the production of anucleate cells—cells devoid of chromosomal DNA—following DNA damage [72, 73]. This RecA-deficient degradation mode is often referred to as “reckless” DNA degradation [74]. We leveraged this phenomenon as a functional assay to test whether interaction between RecB and β-clamp influences the reckless degradation by RecBCD. Specifically, we hypothesized that if β-clamp modulates RecBCD activity, then disrupting their interaction might alter the extent of reckless degradation in a *ΔrecA* mutant background.

A *ΔrecA ΔrecB* strain was transformed with either empty plasmid (pUT18C), pRecB_WT_, pRecB_F1023A_, or pRecB_F489A_. As a positive control for DNA degradation, a *ΔrecA* single-gene deletion strain was included. All strains were cultured to exponential phase (OD_600_ of 0.2), upon which each culture was split into two aliquots: one aliquot was fixed immediately, whereas the second aliquot was exposed to UV irradiation at a dose of 50 J/m^2^. Following UV treatment, cells were allowed to recover for 3 h prior to fixation in ethanol. DNA was stained using Hoechst 33258 and visualized using fluorescence microscopy.

As expected, the *ΔrecA* cells exhibited extensive DNA degradation, with most cells showing little detectable DNA 3 h post-UV (Fig. 7A). Quantitative analysis revealed that *ΔrecA* cells retained only around 16% of the DNA, relative to unchallenged cells (Fig. 7B). The *ΔrecA ΔrecB* double-deletion strain showed a lower reduction in DNA content post-UV (Fig. 7B), retaining ~35%, confirming the role of RecBCD in DNA degradation. Complementation with pRecB_WT_ restored the reckless degradation phenotype, comparable to that of the *ΔrecA* strain (Fig. 7B). Notably, expression of the RecB_F1023A_ mutant reduced DNA degradation compared to wild-type RecB in a highly significant manner (*P* = .0005), whereas RecB_F489A_ did not (Fig. 7B). Introduction of the empty plasmid (pUT18C) into the *ΔrecA ΔrecB* strain did not significantly affect DNA content relative to the uncomplemented mutant.

Under non-damaging conditions, the RecB_F1023A_​ mutant did not significantly alter basal DNA content compared to wild-type RecB (Fig. 7C). These results indicate that the RecB–β-clamp interaction contributes specifically to RecA-independent “reckless” DNA degradation. Since this type of degradation is unusually rapid and extensive, it is possible that the β-clamp functions as a processivity factor for RecBCD in this context—a role not previously described for machineries other than polymerases. However, reckless degradation is likely an aberrant outcome of the absence of RecA, in which the β-clamp remains tethered to RecBCD rather than performing its canonical role in homologous recombination.

Notably, a moderate degree of DNA degradation (∼60%–70%) was observed in Δ*recA* cells even under unchallenged conditions (Fig. 7C), likely reflecting spontaneous fork stalling or collapse requiring RecBCD activity. In the Δ*rec*A Δ*recB* double mutant, most DNA remained intact, consistent with RecBCD being the primary nuclease responsible. Complementation with either wild-type RecB or RecB_F1023A_​ partially restored basal degradation (∼50%), suggesting that complex formation and nuclease activity of RecB_F1023A_ is intact, and that initial processing of DNA ends at stalled or collapsed replication forks (e.g. regressed “chicken foot” structures [31, 32]) can occur independently of β-clamp binding.

## Discussion

In this study, we identified and characterized a novel interaction between the *E. coli* β-clamp and the RecBCD complex. Through a combination of bacterial two-hybrid assays, co-immunoprecipitation, fluorescence microscopy, and NMR spectroscopy, we show that RecB associates with β-clamp via a canonical CBM (QVEMEF) located in the nuclease domain of RecB. Disruption of this motif abolishes binding and compromises cell survival following DNA damage. Functional analyses indicate that the β-clamp–RecBCD interaction is linked to RecBCD conformational switching at Chi sites. These findings expand the β-clamp interactome and implicate β-clamp as a contextual modulator of RecBCD function during the DNA damage response.

### RecBCD associates with β-clamp during unchallenged growth

Using fluorescence microscopy, we showed that around 32% of unchallenged cells displayed RecB and β-clamp foci within proximity of one another, indicating spatial colocalization under normal growth conditions (Fig. 2C and D). Although such proximity cannot be taken as definitive evidence of a direct physical interaction, these observations are consistent with the possibility that RecBCD is located near replication forks or associated structures. This aligns with the view that RecBCD is not exclusively a damage-induced responder but also plays constitutive roles in safeguarding DNA replication fidelity [61].

In unchallenged conditions, β-clamp is largely associated with active replication forks, where it functions as a processivity factor and as a hub for recruiting various enzymes for DNA metabolism. The proximity of RecBCD to β-clamp under these conditions raises the possibility that RecBCD may localize near, or be recruited to, replication forks prior to overt DNA damage. Given that replication forks are frequently challenged by endogenous obstacles—such as DNA secondary structures, tightly bound proteins, or template lesions [62]—it is plausible that RecBCD is recruited in a preemptive or surveillance capacity to help resolve impediments to fork progression. Alternatively, the observed level of relative proximity may reflect replication forks actively undergoing restart or repair. This interpretation is supported by prior evidence in *E. coli* that replication fork stalling occurs multiple times per replication cycle and can lead to fork regression or spontaneous DSB formation [63, 64]. In these contexts, RecBCD is known to engage either RecA-dependent homologous recombination or RecA-independent pathways to facilitate fork restart [61].

Our data indicate that the RecB–β-clamp interaction persists even in a *ΔrecA* background (Supplementary Fig. S1), albeit with delayed detection, suggesting that the interaction does not strictly require the homologous recombination machinery. However, under unperturbed conditions, the RecB_F1023A_ mutation, which abolishes β-clamp binding, retained wild-type proficiency in DNA degradation (Fig. 7C), indicating that β-clamp association is dispensable for basal, RecA-independent processing of regressed forks. These observations suggest that the observed colocalization may primarily reflect spatial proximity at replication forks or a surveillance function rather than a direct role for β-clamp in facilitating degradation under normal growth. The functional relevance of this interaction appears to become pronounced only under conditions of extensive replication stress or DNA damage (see below).

### Implications of RecBCD–β-clamp interactions in response to DNA damage

We also observed colocalization between RecB and β-clamp following ciprofloxacin treatment (Fig. 2D). Although the fraction of colocalizing cells was slightly reduced after treatment, this decrease coincided with a notable reduction in the average number of β-clamp foci per cell (Supplementary Fig. S2B)—likely reflecting widespread replisome disassembly upon drug exposure [65]. Under these conditions, persistent β-clamp foci likely represent β-clamps retained on DNA after replisome collapse, either at sites of unresolved forks or behind previously active replication forks.

Ciprofloxacin at the minimum inhibitory concentration (20 ng/ml) affects the survival of cells in a time-dependent manner through induction of substantial DNA damage [51]. The DNA damage includes replication-dependent and replication-independent DSBs [75, 76], both of which necessitate repair through homologous recombination pathways involving RecBCD and RecA. Although our microscopy data cannot determine whether the RecB–β-clamp interaction occurs at replication forks during DSB repair, at distal damage sites, or primarily under normal growth conditions, additional evidence suggests its role extends beyond DNA replication. First, after UV or ciprofloxacin challenge, we demonstrated 100-fold lowered survival of a strain expressing RecB deficient in β-clamp binding (Fig. 6). Second, we performed an experiment using the BACTH assay, which indicated an increased RecB–β-clamp interaction following UV irradiation (15 J/m^2^) (Supplementary Fig. S3). Third, under the same DNA-damaging condition, the RecB_F1023A_ mutant exhibited markedly reduced “reckless” DNA degradation compared to the wild type, whereas both wild-type and mutated variants were equally proficient under non-damaging growth conditions (Fig. 7). Together, these findings support a model in which RecBCD–β-clamp interactions are not vital for unperturbed replication forks but become functionally important after DNA damage—potentially involving β-clamp retained on DNA after replisome disassembly or at collapsed forks requiring controlled processing.

### Multiple candidate clamp-binding motifs in RecB

We initially identified two candidate CBMs within RecB: QALRF (helicase domain) and QVEMEF (nuclease domain). Only the latter was validated to interact with β-clamp by NMR spectroscopy (Fig. 4), and to affect the functional roles tested here (Figs 6 and 7), though both motifs appeared to support β-clamp interaction in the BACTH system when tested in truncated RecB constructs (Fig. 3C).

It may be that mutations in QALRF disrupt proper helicase domain folding or prevent RecBCD complex assembly, which indirectly leads to loss of interaction. As demonstrated, RecC and RecD subunits are required for detectable interaction in the BACTH assay (Fig. 2A). However, this interpretation is weakened, as shown by the finding that F-to-A substitution within QALRF does not confer a survival defect (Fig. 6), nor affect DNA degradation activity before or after UV exposure (Fig. 7), suggesting that the motif is not essential for core RecBCD function or complex integrity. It may be more likely that the QALRF motif requires specific conformational states, post-translational modifications, or cofactor binding of RecBCD for β-clamp recognition—conditions that are not replicated in the NMR experimental setup. Finally, technical limitations of each method cannot be excluded. STD-NMR is sensitive to interactions within an affinity window of ~10^−8^ M to 10^−3^ M; binding affinities outside this range would escape detection, and the use of CBM peptides lacking native flanking regions may additionally affect the observed binding properties [1]. Conversely, the BACTH signal may represent a false positive arising from proximity-driven adenylate cyclase reconstitution that does not faithfully recapitulate the physiological interaction. Although QALRF is a weaker match to the canonical CBM consensus sequence compared to QVEMEF [1], its similarity to the internal CBM of Pol III (QADMF) suggests possible evolutionary or structural relevance. Given that Pol III possesses both internal and C-terminal CBMs [2, 12, 68] it is plausible that RecB also employs a dual-motif architecture or that other regions not identified here participate in and stabilize the interaction. Indeed, multiple binding surfaces have been described on β-clamp, allowing for simultaneous or sequential interaction with various client proteins [77–79]. It is therefore conceivable that RecB, either alone or in the context of the RecBCD complex, engages the clamp via additional, noncanonical interfaces or transient contacts that escape detection by motif searches or *in vitro* assays. Further investigation, including structural and mutational analyses beyond canonical CBM sequences, will be needed to fully map the interaction interface.

The QVEMEF motif aligns well with key features of known CBMs, including a conserved glutamine at position 1, an aliphatic residue at position 2, a methionine at position 4, and an essential phenylalanine at the last position. Its validation by NMR and its functional importance in β-clamp binding and nuclease activity underscore its biological relevance.

### Conformational regulation of RecBCD and the role of the QVEMEF motif

The QVEMEF motif resides within the nuclease domain of RecB, a region buried in the crystal structure of the DNA-bound RecBCD complex (Fig. 3B). This positioning suggests that β-clamp interaction via this motif requires structural rearrangement. According to the swing model [80], Chi recognition triggers a dramatic conformational shift in RecBCD: the RecB nuclease domain swings outward on its 19 amino-acid linker—moving from its initial tucked position to emerge near the RecC tunnel exit—thereby potentially exposing motifs such as that involved in β-clamp binding and enabling the switch from DNA degradation to recombination initiation.

Recent structural and biochemical evidence complicates this model. Pavankumar *et al*. [81] demonstrated that the nuclease domain remains in its pre-Chi conformation until the 5′ DNA tail exceeds ~10 nt. Only then do more extensive conformational shifts occur—likely involving partial loosening rather than full domain rotation. These intermediate states appear sufficient to support RecA loading and modulate enzymatic activity without full domain detachment.

In this context, exposure of the QVEMEF motif may be dynamically regulated by DNA substrate structure and Chi recognition. Such conditional accessibility could modulate β-clamp binding, potentially stabilizing a recombination-competent conformation or tuning RecBCD activity in response to replication stress or specific DNA intermediates. Current structural models of RecBCD are based on its DNA-bound conformation, where the RecB nuclease domain is buried and the QVEMEF motif is inaccessible. However, β-clamp may bind RecBCD in a DNA-free or partially unbound state—such as at free DNA ends or prior to re-engagement with a substrate—where alternative conformations expose interaction motifs. Since no structures of the full, unbound RecBCD complex exist, it may be that β-clamp binding reflects a conformation distinct from those captured crystallographically, possibly inducing or stabilizing a recombination-ready state.

Our BACTH data support direct interaction between the QVEMEF motif and β-clamp (Fig. 3C), as mutation of the motif abolishes the interaction. NMR analysis further confirms this interaction and shows that a single F1023A substitution is sufficient to disrupt binding (Fig. 4C and D). Several residues in QVEMEF—particularly valine and the two glutamates—have been implicated in RecA binding [29], but the phenylalanine has not. Additionally, a RecB mutation (D1080A), which disrupts the interaction between RecB and RecA, has been demonstrated to lead to survival rates after UV exposure that are comparable to those observed in strains with a complete *recB* deletion [82]. Importantly, we tested the ability of RecB_F1023A_ to bind RecA, which was identical to that of RecB_WT_ (Supplementary Fig. S8). Thus, the observed, moderate survival defect (Fig. 6) is unlikely to result from impaired RecA interaction.

Structural models suggest that F1023 may also contact RecC in the pre-Chi conformation [24]. Although we cannot fully exclude that the RecB F1023A substitution partially affects catalytic activity or the RecB–RecC contact, several observations suggest that its nuclease function remains largely intact. Under unperturbed growth, cells lacking both *recA* and *recB* exhibited elevated DNA content, whereas complementation with either wild type RecB or RecB_F1023A_ restored DNA degradation to comparable levels (Fig. 7C). In contrast, following UV or ciprofloxacin exposure, RecB_F1023A_ cells displayed diminished DNA degradation (Fig. 7B) and markedly reduced survival (Fig. 6). Together, these results confirm that nuclease activity is intact and additionally indicate that β-clamp binding is dispensable for the basal degradation of regressed forks (“chicken-foot” structures [31, 32]).

For these reasons, β-clamp is likely not a general cofactor for RecBCD activity. Instead, the most plausible scenario is that β-clamp becomes functionally relevant *after* RecBCD encounters a Chi site. At this transition point, RecBCD shifts from extensive degradation to generating the 3′ ssDNA tail needed for RecA loading and recombination. We propose that β-clamp is recruited at or near this stage to help assemble the replisome once RecA has completed strand invasion and replication restart must occur.

The Δ*recA* data support this interpretation: in the absence of RecA, β-clamp remains associated with RecBCD and appears to act—aberrantly—as a processivity factor during the hyperactive “reckless degradation” phenotype. If β-clamp can indeed serve as a processivity factor for a nuclease–helicase complex other than a DNA polymerase, this would represent a previously unrecognized and mechanistically unusual role for the clamp. However, such usage is likely a pathological consequence of lacking RecA rather than a normal physiological function.

In a wild-type context, we therefore infer that RecA is required to position β-clamp correctly—either before or after strand invasion—so that the clamp is handed off to its proper function in replication restart/DNA re-synthesis rather than remaining tethered to RecBCD. This model links our biochemical finding (the CBM-mediated β-clamp interaction), our functional data (altered “reckless” DNA degradation phenotypes), and known RecBCD–Chi–RecA transitions into a coherent mechanism that does not necessarily invoke broader or unsupported roles for β-clamp in recombination.

Comparative considerations between RecB and eukaryotic homologs further highlight the evolutionary conservation of clamp–nuclease coordination. In eukaryotes, DNA end resection and fork restart are mediated by the Mre11–Rad50–Nbs1 (MRN) complex and the RecQ–DNA2–EXO1 pathways, which functionally parallel bacterial RecBCD [83–85]. Several of these eukaryotic components, including EXO1 and Werner syndrome helicase (WRN), directly interact with the sliding clamp PCNA through a canonical PCNA interacting protein-box or related motifs, facilitating their recruitment to stalled forks [86, 87]. In contrast, the MRN complex and Bloom syndrome protein (BLM) helicase act upstream or in association with PCNA-bound factors but lack direct clamp binding [88, 89]. This suggests that physical association with the clamp is not universally required for DNA end processing but may have evolved as a modular mechanism to couple resection and repair with replisome components. In this light, the RecB–β-clamp interface may represent an ancestral prototype of such regulatory connections between clamp loaders and DNA repair machineries.

Our findings illuminate the multifunctionality of the sliding clamp, expanding its role in coordinating replication and repair pathways during the bacterial DNA damage response. Disrupting the RecB–β-clamp interaction could present novel targets for antimicrobial intervention. Further work is needed to resolve the structural details and assess the physiological relevance of the interaction of β-clamp with RecBCD.

## Supplementary Material

gkag570_Supplemental_File

## Data Availability

Data underlying this article will be shared on reasonable request to the corresponding author. The MicrobeJ template used for analyzing colocalization between RecB and β-clamp in this study is available in the Zenodo repository at https://doi.org/10.5281/zenodo.19152453.

## Notes

Present address: Human Health Ingredients AS, Aker BioMarine, 1327 Lysaker, Norway
