# Supplementary material for: The RecBCD complex interacts directly with the DNA sliding clamp in Escherichia coli
Source: Nucleic Acids Res. 2026 Jun 11;54(11):gkag570. doi: 10.1093/nar/gkag570 (PMC13254535; doi:10.1093/nar/gkag570)
Supplement: gkag570_Supplemental_File [file gkag570_supplemental_file.pdf]

SUPPLEMENTARY DATA

The RecBCD complex interacts directly with the DNA sliding clamp in *Escherichia coli*

Ida Mathilde Marstein Riisnæs[1,2,†], Synnøve Brandt Ræder[1,†], Signe Simonsen[3], Krister Vikedal[1,2], Paul Hoff Backe[1,4], Line Johnsen[1,‡], Magnar Bjørås[1,2,5], James Alexander Booth[1,5], Birthe B. Kragelund[3], Kirsten Skarstad[1] and Emily Helgesen[1,5,*]

[1] Department of Microbiology, Oslo University Hospital, Rikshospitalet, 0373 Oslo, Norway
[2] Department of Microbiology, University of Oslo, 0316 Oslo, Norway
[3] Structural Biology and NMR Laboratory, Department of Biology, University of Copenhagen, 2200 Copenhagen
[4] Department of Medical Biochemistry, Institute of Clinical Medicine, University of Oslo, 0372 Oslo, Norway
[5] Department of Clinical and Molecular Medicine, Norwegian University of Science and Technology, 7030 Trondheim, Norway.

† Co-first authors
‡ Current affiliation: Aker BioMarine, Human Health Ingredients AS, 1327 Lysaker, Norway
* To whom correspondence should be addressed. Email: Emily.helgesen@ous-research.no

Table S1. Hit *E. coli* genes from the yeast two-hybrid screening, classified according to the confidence of their interaction with the β-clamp (*dnaN*). The cellular localization of the gene products is listed. The *recB* hit is highlighted.

Gene	Confidence in interaction	Cellular localization
amiC	Moderate	Cytoplasm
b0942	Moderate	Membrane
b1504	High	Membrane
b1978	Very high	Membrane
b2074	Very high	Membrane
b2075	Good	Membrane
b2225	Very high	Membrane
b2520	Moderate	Membrane
b2973	High	Membrane
bglX	Very high	Membrane
cysI	High	Cytoplasm
dnaX	Moderate	Cytoplasm
eno	Very high	Membrane
fecA	Good	Membrane
ftsK	High	Cytoplasm
fusA	High	Cytoplasm
infA	Good	Membrane

**Gene**	**Confidence in interaction**	**Cellular localization**
ligA	High	Cytoplasm
lipB	Moderate	Cytoplasm
mepA	Moderate	Membrane
modA	Moderate	Membrane
nmpC	Very high	Membrane
ompC	Very high	Membrane
ompN	Very high	Membrane
pepA	High	Cytoplasm
pepT	Good	Cytoplasm
pflA	Good	Cytoplasm
phoE	Very high	Membrane
ppsA	High	Cytoplasm
prpD	Moderate	Cytoplasm
recB	**Moderate**	**Cytoplasm**
rpe	Good	Cytoplasm
secD	Moderate	Membrane
speB	Very high	Cytoplasm
sufI	Good	Membrane
talc	High	Cytoplasm
tdh	Moderate	Cytoplasm
tolA	High	Membrane
torC	High	Membrane
yadM	Good	Membrane
yagX	Moderate	Membrane
ydbA_1	Good	Membrane
yehB	Moderate	Membrane
ytfN	Very high	Membrane
yhjN	Very high	Membrane
yihF	Moderate	Membrane
yjjQ	Good	Cytoplasm

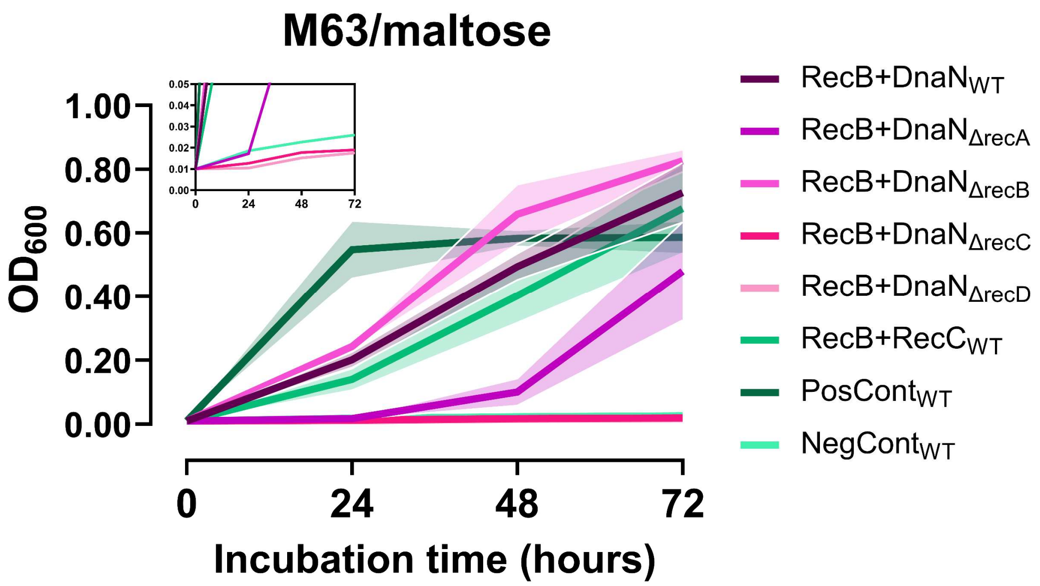

Figure S1. BACTH-based interaction between RecB and β-clamp in wild-type and RecBCD mutant backgrounds. Growth of *E. coli* BTH101 strains co-expressing RecB–T18 and β-clamp (DnaN)–T25 fusion proteins was monitored in M63/maltose minimal medium over 72 hours at 30 °C. OD600 was measured at 24, 48, and 72 hours to assess interaction-dependent growth based on reconstitution of adenylate cyclase activity. The RecB–β-clamp interaction was assessed in wild-type BTH101 and in isogenic deletion strains lacking *recA*, *recB*, *recC*, or *recD*. Wild-type cells co-expressing RecB and RecC were included as an internal control for protein–protein interaction. Co-transformed *zip* fusions served as a positive control, and empty vectors (T18 and T25 alone) as a negative control. While interaction-dependent growth was observed in wild-type, *ΔrecA*, and *ΔrecB* strains, the interaction was strongly impaired in both *ΔrecC* and *ΔrecD* backgrounds—suggesting a requirement for intact RecBCD architecture. Lines represent means from three biological replicates; shaded regions indicate SEM. Inset (top left): the y-axis is adjusted (OD 0–0.05) to better resolve strains exhibiting very slow growth.

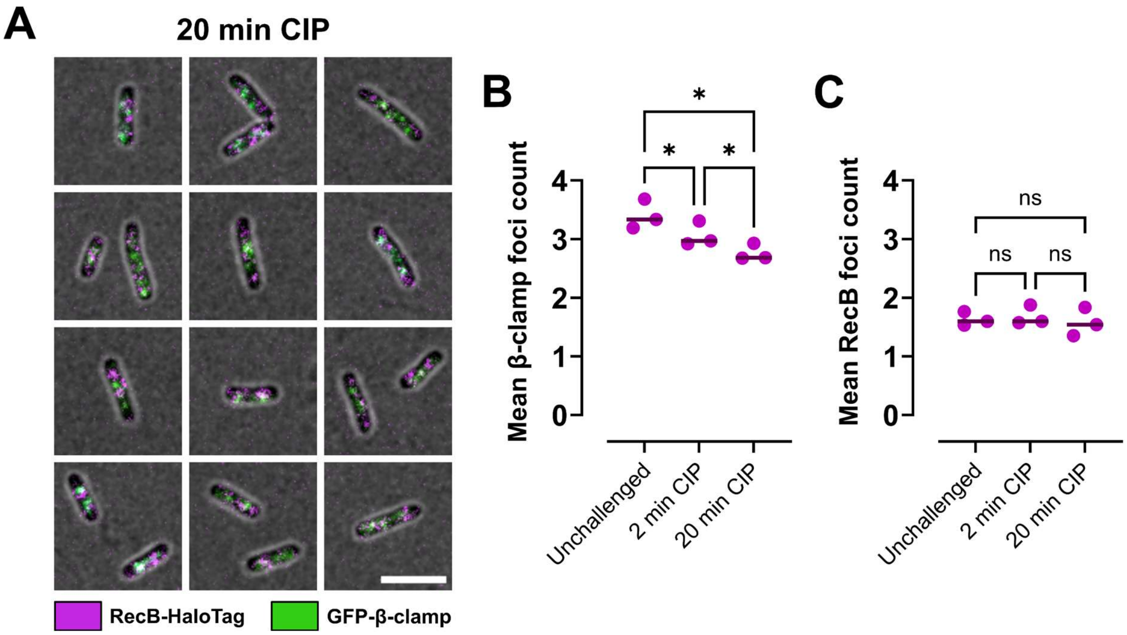

Figure S2. Ciprofloxacin treatment reduces β-clamp foci but not RecB foci in live *E. coli* cells. (**A**) Representative fluorescence microscopy images of fixed EH219 cells treated with ciprofloxacin (20 ng/mL, 20 min), showing JF549-labeled RecB–HaloTag (magenta) and endogenous GFP–β-clamp (green). Colocalized signals appear white. (**B**) Quantification of mean β-clamp foci per cell in untreated cells and after 2 or 20 min of ciprofloxacin treatment. (**C**) Quantification of mean RecB foci per cell under the same conditions. Statistical comparisons were made using repeated measures one-way ANOVA with Tukey's multiple comparisons test. Lines represent the mean of three biological replicates; dots represent individual replicate means. ns, not significant; *$P \leq 0.05$.

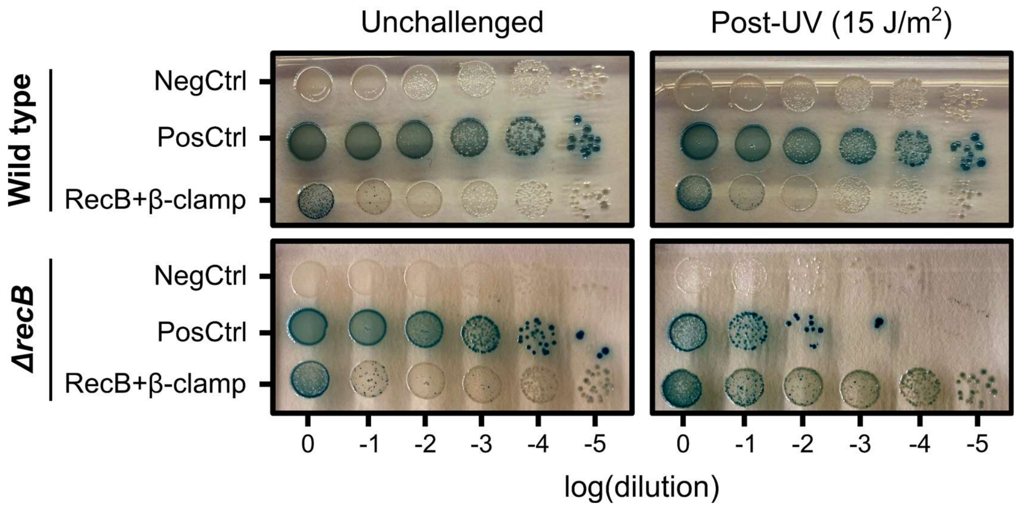

Figure S3. UV-induced enhancement of the RecB–β-clamp interaction detected by blue-white assay. Representative blue-white spot assay assessing interaction between RecB–T18 and β-clamp (DnaN)–T25 fusion proteins in wild-type (BTH101, upper panel) and *ΔrecB* (BTH101 *ΔrecB*, lower panel) backgrounds, with (left panels) or without (right panels) UV irradiation (15 J/m^2). Empty vector (negative) and leucine zipper (*zip*; positive) controls were included. The assay suggests that the RecB–β-clamp interaction is strengthened following UV-induced DNA damage, particularly in the absence of endogenous RecB.

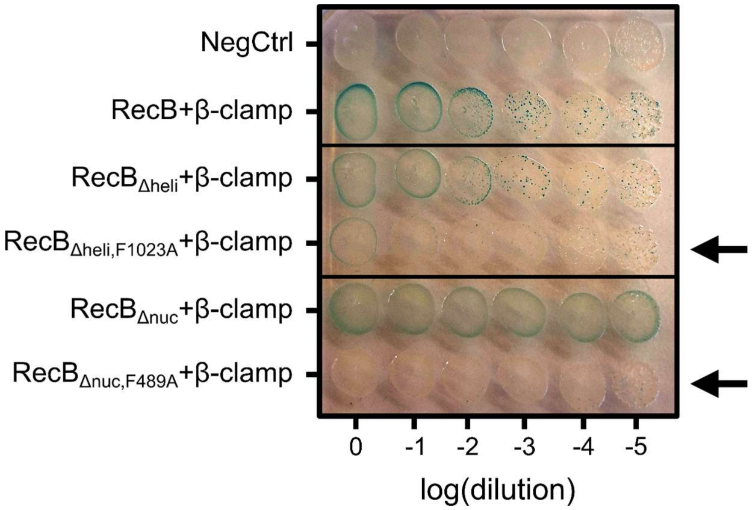

Figure S4. Analysis of truncated RecB variants and β-clamp interaction via bacterial two-hybrid blue-white assay. Interaction between the β-clamp and truncated RecB variants lacking either the helicase domain (RecB$_{\Delta heli}$) or the nuclease domain (RecB$_{\Delta nuc}$) was assessed by blue-white screening. Single alanine substitutions F1023A (in RecB$_{\Delta heli}$) and F489A (in RecB$_{\Delta nuc}$) resulted in a markedly reduced interaction compared to their respective non-mutated truncated counterparts, as indicated by decreased blue pigment intensity (arrows). Cells co-transformed with empty pUT18C and pKT25 vectors served as the negative control, while full-length RecB and β-clamp co-expression served as the positive control.

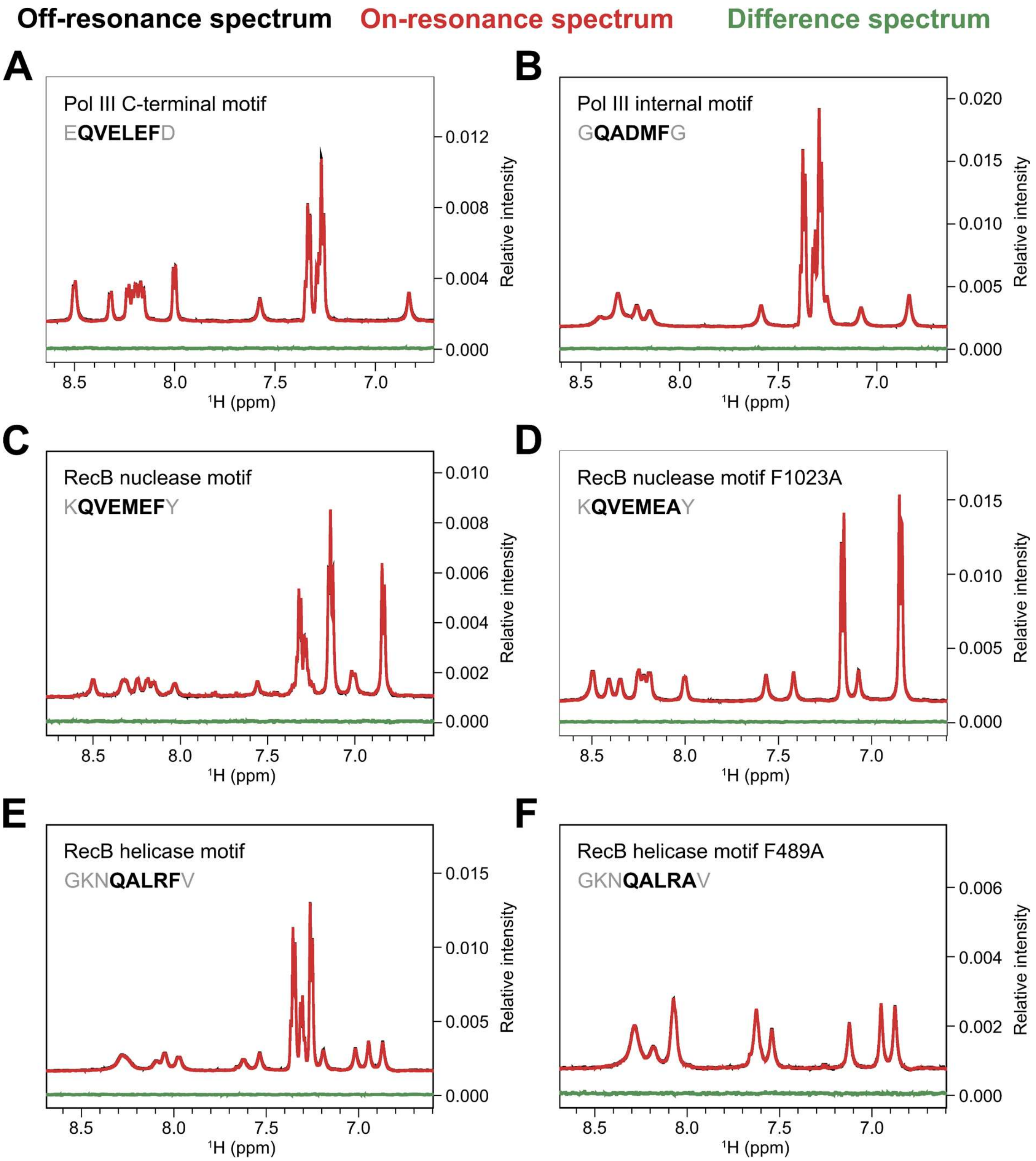

Figure S5. Control STD-NMR spectra confirm that peptides do not experience saturation in the absence of β-clamp. (**A-F**) Saturation Transfer Difference (STD) NMR spectra of synthetic peptides in the absence of purified β-clamp protein. For each sample, the off-resonance (reference) spectrum is shown in black, the on-resonance (saturated) spectrum in red, and the resulting STD difference spectrum in green. Peptides tested: (A) EQVELEFD, (B) GQADMFG, (C) KQVEMEFY, (D) KQVEMEAY, (E) GKNQALRFV, and (F) GKNQALRAV. No detectable signals were observed in the difference spectra under the conditions used, confirming that the peptides do not experience saturation in the absence of β-clamp binding.

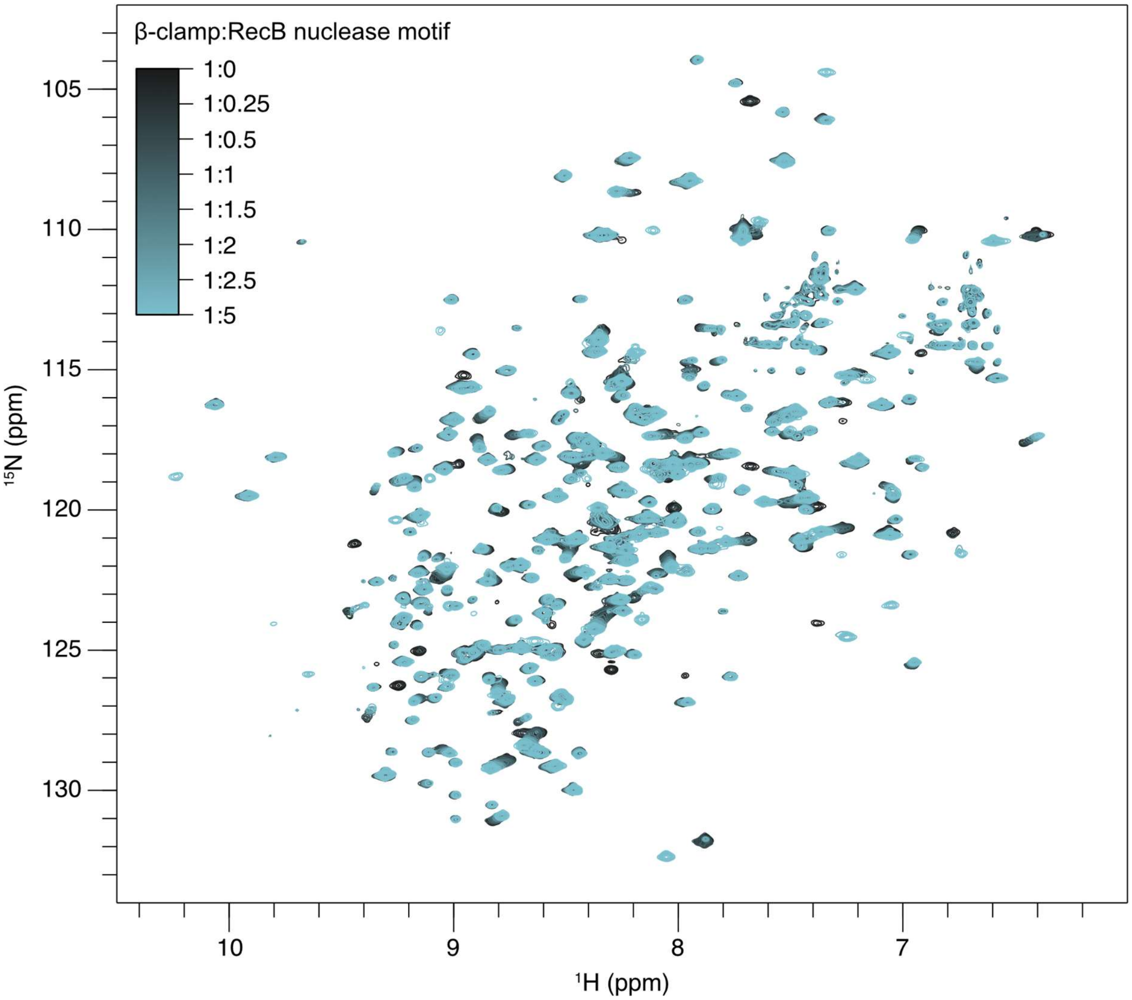

Figure S6. ^{1}H,^{15}N TROSY-HSQC titration of ^{2}H,^{13}C, ^{15}N-labelled β-clamp with the addition of the RecB nuclease motif (QVEMEF) at different molar ratios. It is possible that the peptide aggregates at the highest concentrations used, explaining why a high molar excess of the RecB nuclease motif is needed to fully saturate β-clamp.

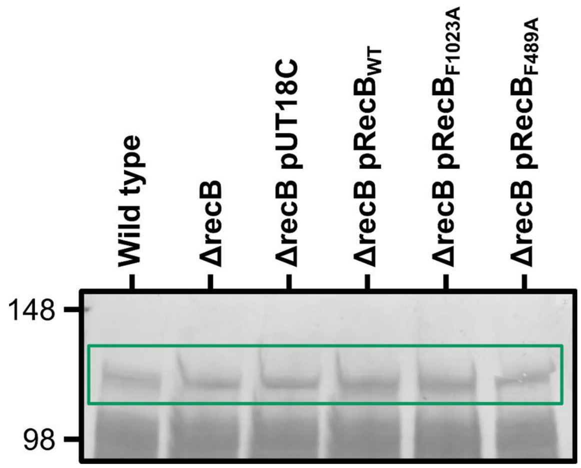

Figure S7. Ponceau S staining of total protein for normalization of western blot quantification. Ponceau S staining of the membrane used for western blot analysis (Figure 6C) confirms approximately equal loading of total protein across all lanes. The membrane was stained following transfer and prior to blocking and immunodetection. The indicated area was used to normalize band intensities for quantification of protein expression shown in Figure 6D.

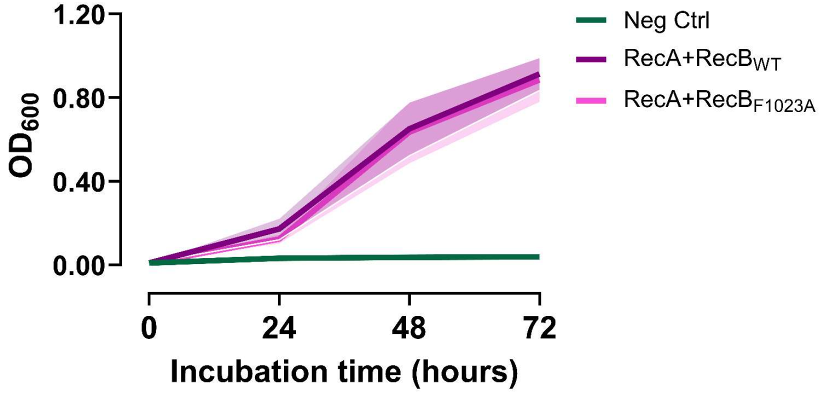

Figure S8. BACTH-based interaction between RecA and RecB variants in M63/maltose minimal medium. Growth of *E. coli* BTH101 strains co-expressing RecA–T25 and RecB–T18 fusion proteins was monitored in M63/maltose minimal medium over 72 hours at 30 °C. OD_{600} was measured at 24, 48, and 72 hours to assess interaction-dependent growth based on reconstitution of adenylate cyclase activity. Co-expression of RecA with wild-type RecB ($RecA+RecB_{WT}$) or the $RecB_{F1023A}$ mutant ($RecA+RecB_{F1023A}$) resulted in comparable interaction-dependent growth, indicating that the F1023A substitution does not impair the RecA–RecB interaction. Empty vectors (T18 and T25 alone) served as negative control. Lines represent means from three biological replicates; shaded regions indicate SEM.
